# NetG2P: Network-based genotype-to-phenotype transformation identifies key signaling crosstalk for prognosis in pan-cancer study

**DOI:** 10.1186/s12915-026-02559-x

**Published:** 2026-02-24

**Authors:** Jonghyun Lee, Seok-Won Jang, Byungjo Lee, Jisu Shin, Jeong-Ryeol Gong, Dongkwan Shin

**Affiliations:** 1 Research Institute, National Cancer Center, Goyang, 10408 Republic of Korea; 2 Deparment of Biochemistry and Molecular Genetics, University of Virginia School of Medicine, Charlottesville, VA 22903 USA; 3 Department of Genome Sciences, University of Virginia School of Medicine, Charlottesville, VA 22903 USA; 4 Department of Bio and Brain Engineering, Korea Advanced Institute of Science and Technology (KAIST), Daejeon, 34141 Republic of Korea; 5 Department of Cancer Biomedical Science, National Cancer Center Graduate School of Cancer Science and Policy, Goyang, 10408 Republic of Korea

**Keywords:** Genotype-to-phenotype transformation, Signaling crosstalk, Critical oncogenic feature, Network propagation, Machine learning, Drug repurposing, Pan-cancer analysis

## Abstract

**Background:**

Despite advances in whole-genome sequencing and identifying cancer-associated genetic alterations, understanding the influence of multiple genetic alterations collectively on cancer phenotypes remains challenging, owing to mutation pattern complexity and variability. Here, we present the NETwork-based Genotype-to-Phenotype Transformation (NetG2P), which utilizes network propagation to translate genomic information into pathway interaction networks. Using the Cancer Genome Atlas dataset across 10 cancer types, we conducted a pan-cancer analysis using NetG2P to uncover critical oncogenic features associated with cancer prognosis using machine learning and explainable artificial intelligence models.

**Results:**

Our results suggest that these features, which primarily represent signaling crosstalk, can serve as functional units for determining cancer prognosis. Network analysis of these critical oncogenic features reveals distinct patterns among cancer types, categorizing them into “distributed” and “modular” networks based on pathway interactions. Applying this technique to cancer cell line data has helped predict novel drug targets for high-risk groups and proposed candidates for drug repurposing.

**Conclusions:**

NetG2P generates patient-specific networks of critical oncogenic features and suggests personalized treatments, hence advancing precision medicine in oncology.

**Supplementary Information:**

The online version contains supplementary material available at 10.1186/s12915-026-02559-x.

## Background

Whole-genome sequencing has significantly advanced our ability to investigate cancer-associated genetic alterations, including identifying driver mutations. For instance, KRAS mutations are associated with poor prognosis in lung and colorectal cancers [1, 2], whereas BRCA gene family mutations are associated with a higher risk of developing breast and ovarian cancers [3, 4]. Numerous studies on cancer mutation patterns have investigated the interactions between multiple driver mutations, such as co-occurring and mutually exclusive mutation pairs [5, 6]. These interactions provide insight into the synergistic effects that promote cancer development and the synthetic lethality that can be exploited for cancer therapies [7]. However, despite these efforts, elucidating the influence of multiple genetic alterations on tumor progression, cancer phenotypes, and therapeutic responses remains a formidable challenge. Mutation patterns are complex and variable, creating significant obstacles in establishing clear genotype–phenotype relationships in cancer [8].

Biological networks can assist in understanding the widespread effects of small gene subset alterations on complete cellular processes [9]. Recent bioinformatics studies have proposed network analysis approaches based on random walks and diffusion processes, known as network propagation [10–16]. This approach integrates multiple information sources and mutations in genetic disease to achieve patient stratification, identify disease subtypes and modules, and predict novel drug targets. When applied to cancer, network propagation transforms genetic alterations into oncogenic signaling pathways. Recently, Elmarakeby et al. merged artificial intelligence (AI) with biological pathways to determine biological functions that were most significantly affected in prostate cancer [17]. These pathway-based analyses enable a more comprehensive interpretation of genomic data compared to interpretations at the genomic level, and assist in developing new diagnostic tools and discovering novel prognostic markers and anticancer drug targets.

Biological pathways are not isolated, but interconnected with each other, collectively contributing to cellular phenotypes. Signaling crosstalk, defined as the shared components between two or more pathways, plays a crucial role in signal integration, simultaneous activation of multiple pathways, and potential reactivation of inhibited pathways via bypassing blocked elements [18, 19]. Therefore, understanding the coordination between pathways through crosstalk is essential for developing effective cancer treatments. Recent studies have highlighted the significance of signaling crosstalks in tumor heterogeneity and treatment responses [20, 21]. These insights have led to the development of novel therapeutic strategies that leverage key signaling pathways and their crosswalks in cancer [18]. However, to our knowledge, no systematic pan-cancer approach has been developed that utilizes multiple oncogenic pathways and their crosswalks as functional biological units to describe critical functions in human cancer cells. Therefore, defining cancer characteristics at the pathway crosstalk level is necessary for developing more effective combination therapies.

Here, we present a network-based genotype-to-phenotype transformation (NetG2P), designed to translate the genomic information of patients with cancer into pathway interaction networks via involving network propagation and explainable AI (XAI). We conducted a pan-cancer analysis using NetG2P on The Cancer Genome Atlas (TCGA) dataset to identify critical oncogenic features (COFs) and sets of cancer prognosis-associated pathways and their signaling crosstalk, across 10 cancer types. Our results demonstrate that applying network propagation to mutation profiles and incorporating signaling crosstalk with oncogenic pathways significantly improves model performance, highlighting the importance of interactions among multiple oncogenic pathways in determining cancer prognosis. The pan-cancer analysis revealed distinct patterns of the pathway interaction networks among different cancer types. For instance, bladder cancer (BLCA), uterine corpus endometrial carcinoma (UCEC), and ovarian cancer (OV) exhibited a “distributed” network, wherein COFs affected multiple cancer hallmarks. In contrast, stomach adenocarcinoma (STAD) and liver hepatocellular carcinoma (LIHC) displayed a “modular” network, with COFs influencing one or two cancer hallmarks. NetG2P was applied to perturbation data from cancer cell lines and it successfully predicted novel drug targets for prognostically higher-risk groups and proposed drug candidates for repurposing. Our approach involves constructing patient-specific networks of COFs, which are crucial in facilitating personalized treatment approaches as a strategy in precision medicine.

## Results

### Overview of NetG2P

**Fig. 1:**
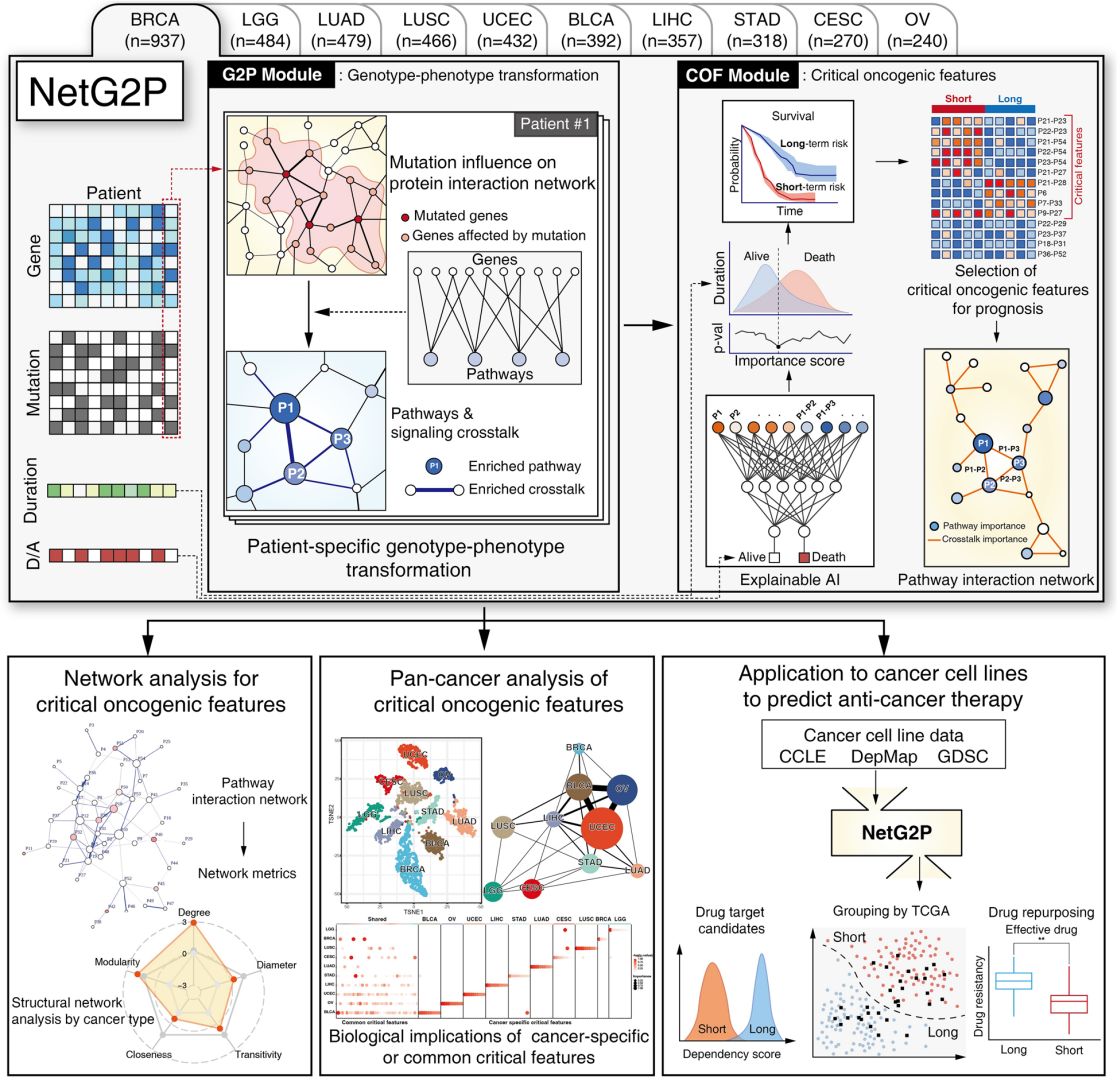
An overview of network-based genotype-to-phenotype transformation (NetG2P) and its applications. Using COFs, we demonstrate that the structural organization of signal crosstalk itself is significant, in which biological functions are impaired in different cancer types. Comparing COFs from different cancer types reveals similarities between previously unassociated cancers and pathways with frequent crosstalk in cancer progression. Utilizing perturbation data from cancer cell lines facilitates the identification of potential drug targets and drug repurposing candidates

In this study, we define genotype as the patient-specific somatic mutation profile (DNA-level alterations). We define phenotype as the patient-specific functional state of oncogenic pathways and their signaling crosstalk, represented as the oncogenic feature matrix derived through network propagation. Gene expression data are incorporated as contextual information to estimate the functional impact of somatic mutations during this propagation. Clinical survival outcome is subsequently used as an external endpoint to identify prognostically relevant components of this phenotypic state, which we refer to as critical oncogenic features (COFs). NetG2P comprises the G2P and COF modules (Fig. 1, top panels). The G2P module uses gene expression and mutation data from individual patients with cancer as input to identify the largest cluster within a protein interaction network likely influenced by multiple somatic mutations. This cluster is then analyzed for significant enrichment in 54 cancer-related biological pathways and their crosstalk, thus forming a patient-specific pathway interaction network. These enriched pathways and their crosstalk are designated as oncogenic features. This oncogenic feature per cancer patient matrix is passed to the COF module.

The COF module aims to identify oncogenic features critical for cancer prognosis from the oncogenic feature matrix from the G2P module. The employed machine-learning models and XAI approach to integrate oncogenic features of all TCGA patients with cancer determines the importance scores of oncogenic features in predicting the vital status of patients with cancer (see Methods for details). Survival analysis determined the optimal threshold for oncogenic features that effectively stratified the patients into two groups with significantly different prognoses. This subset of oncogenic features was designated as COF, and the resulting patient groups were classified as short- and long-term risk groups based on their relative prognosis. We applied this analysis across 10 cancer types, extracting COFs for each type (Additional file 1: Table S1) and constructing cancer type-specific pathway networks of COFs (Additional file 2: Figure S1).

COF identification provides a comprehensive understanding of the key pathways and their interactions that guide prognosis across different cancer types (Fig. 1, bottom panels). Pan-cancer analysis revealed both common and cancer type-specific mechanisms underlying cancer progression and patient outcomes. The networks formed by these COFs were structurally analyzed using several network metrics associated with information flow, which suggested distinct patterns of pathway interactions across cancer types. Finally, we extended NetG2P to a cancer cell line dataset, predicting potential drug targets and identifying existing drugs that could be repurposed for short-term risk groups.

### NetG2P reveals COFs distinguishing short-term and long-term cancer risk groups

**Fig. 2:**
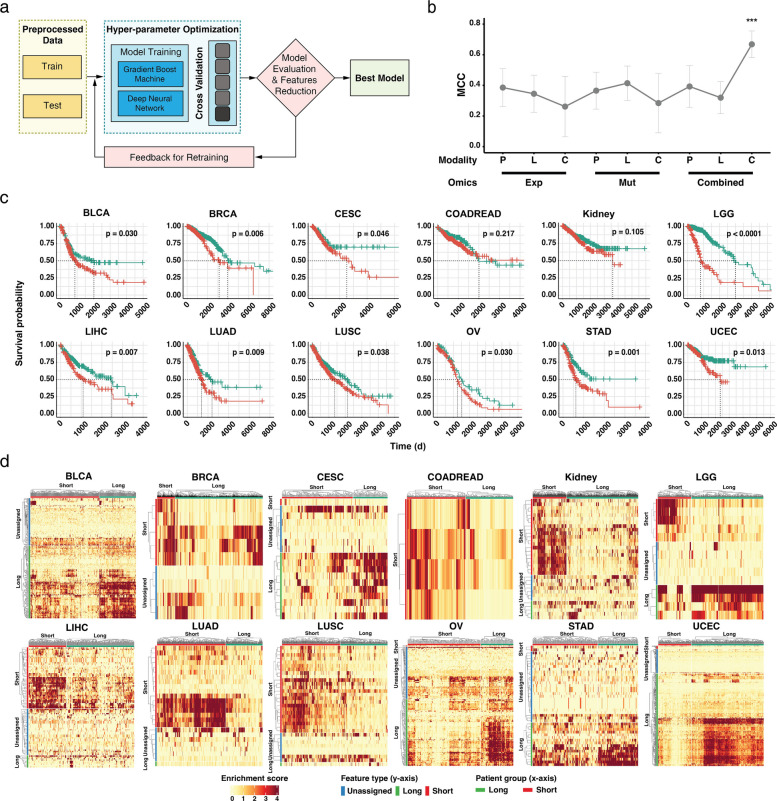
Network-based genotype-to-phenotype transformation (NetG2P) model training, performance evaluation, and survival analysis of cancer datasets. **a** Schematic representation of the model training in the NetG2P pipeline. Preprocessed data is divided into training and test sets, which is hyper-parameter optimized. Two models, gradient boost machines and deep neural networks, are trained, validated, and retrained with feature reduction techniques to select the best model. **b** Performance comparison of ablation study. Prediction performance was evaluated using the Matthews correlation coefficient (MCC). Performance was measured on either single-omics (Exp: expression, Mut: mutation), with different sets of oncogenic features (P: Pathway-only, L: Pathway-links, C: Combined). The highest performance is achieved with the integration and full set of oncogenic features (***p < 0.001). **c** Kaplan-Meier survival curves for 12 cancer types and their short- and long-term risk groups separated by critical oncogenic features (COFs). With the exception of KIDNEY and COADREAD, all risk groups met the statistical significance. **d** Heatmaps displaying the enrichment scores of COFs across different cancer types. COF type is shown on the *y*-axis and patient group is indicated on the *x*-axis, with the red color representing higher enrichment scores

The G2P module integrates gene expression and somatic mutation data from cancer patients to generate an oncogenic feature matrix for each cancer type. This matrix represents patient-specific enrichment scores across 54 oncogenic signaling pathways and their crosstalk (see Methods for details). This transformation provides a clear interpretation of cellular functions compared to evaluating genomic information alone. To further refine the oncogenic features that impacts prognosis, we developed a model using a machine-learning pipeline to predict the prognosis based on oncogenic feature matrix (Fig. 2a). Genotype-to-phenotype transformation significantly reduced input data complexity from 60,660 genes into 1485 oncogenic features, which simplified machine learning models. This, in turn, aided in applying XAI techniques effectively. The model was trained on TCGA cohorts and the highest-performing model was selected for each cancer type.

We evaluated the model performance using ablation analysis and comparisons with other omics-based models (Fig. 2b). Our G2P model, which utilized the network propagation and a complete list of oncogenic features, achieved an average Matthews correlation coefficient (MCC) of 0.668 across 12 cancer types. In contrast, models using individual omics data without considering network propagation showed a significantly lower performance, with average MCCs of 0.262 (expression-only) and 0.285 (mutation-only) (Fig. 2b and Additional file 1: Table S2). Models considering only 52 pathways or 1431 pathway links achieved average MCCs of 0.393 and 0.320, respectively. Crucially, removing any single component from the G2P module drastically reduced prediction performance. In the one-versus-all model performance comparisons across individual cancer types, NetG2P achieved the highest MCC in 11 of the 12 cancer types (Additional file 1: Table S2). These results demonstrate that considering signaling crosstalk via network propagation of multiple somatic mutations is essential for accurately predicting cancer prognosis. We benchmarked NetG2P against other network- and pathway-based approaches to validate its performance (see Methods for details). Across these benchmarks, NetG2P demonstrated the strongest performance among the compared methods in predicting patient vital status (Additional file 2: Figure S2 and Additional file 1: Table S3).

To reduce heterogeneity due to diverse anticancer treatment regimens, we evaluated NetG2P’s prognostic stratification within TCGA subsets receiving the same drug (see Methods for details). Prognostic separation remained significant across all 8 evaluable cancer–drug pairs (Additional file 2: Figure S3 and Additional file 1: Table S4).

To assess generalizability beyond TCGA, we additionally validated NetG2P on independent cohorts from LinkedOmics and ICGC [22, 23] (see Methods for details). We observed significant survival stratification in CPTAC-LSCC (*p* = 0.012) and ICGC-LIRI-JP (*p* = 0.026), with a consistent trend in CPTAC-LUAD (*p* = 0.064) (Additional file 2: Figure S4 and Additional file 1: Table S5).

Using XAI, we extracted the relative importance of all oncogenic features from the best-performing model (see Methods). Optimal threshold for filtering COFs were obtained from the survival analysis with the lowest *p*-value (Fig. 2c). We extracted COFs from 10 cancer types ranging from 9 (for BRCA) to 447 (for UCEC) (Additional file 1: Table S1, Table S6). In total, 617 unique COFs were identified across all cancer types. COFs shared between at least two cancer types were more common than cancer-type specific COFs (395 shared vs. 222 cancer-specific). The oncogenic pathway with the most crosstalk, normalized by pathway size, was P54 (nuclear factor (NF)-κB signaling), followed by P18 (adherens junction), P48 (HIF-1 signaling), P15 (p53 signaling), and P12 (TGF-β signaling) (Additional file 1: Table S7). The high frequency of pathway crosstalk involving NF-κB signaling as a critical feature was consistent with its diverse functions implicated in tumor cells during cancer progression [24–28]

COFs were further classified into 112 short-term and 699 long-term COFs based on their statistical association with the risk groups. A heatmap of the COF revealed that short- and long-term COFs generally clustered well within their respective risk groups, although the patterns varied across cancer types (Fig. 2d). For instance, LUAD showed a near-exact match between the short-term COFs and risk group, whereas the patterns in other cases were less clear (long-term COFs of STAD) or only partially enriched (short-term COFs of LGG or long-term COFs of UCEC). The COF distribution also varied; LGG showed a balance between both types, whereas BLCA, CESC, OV, STAD, and UCEC were dominated by long-term COFs and the remaining cancer types had more short-term COFs. In summary, NetG2P successfully stratified patients with cancer into risk groups using COFs; therefore, it offers interpretable prognostic predictions compared to traditional genomic analyses and provided valuable insights into potential intervention targets.

### Pan-cancer analysis reveals shared and unique COFs across cancer types

**Fig. 3:**
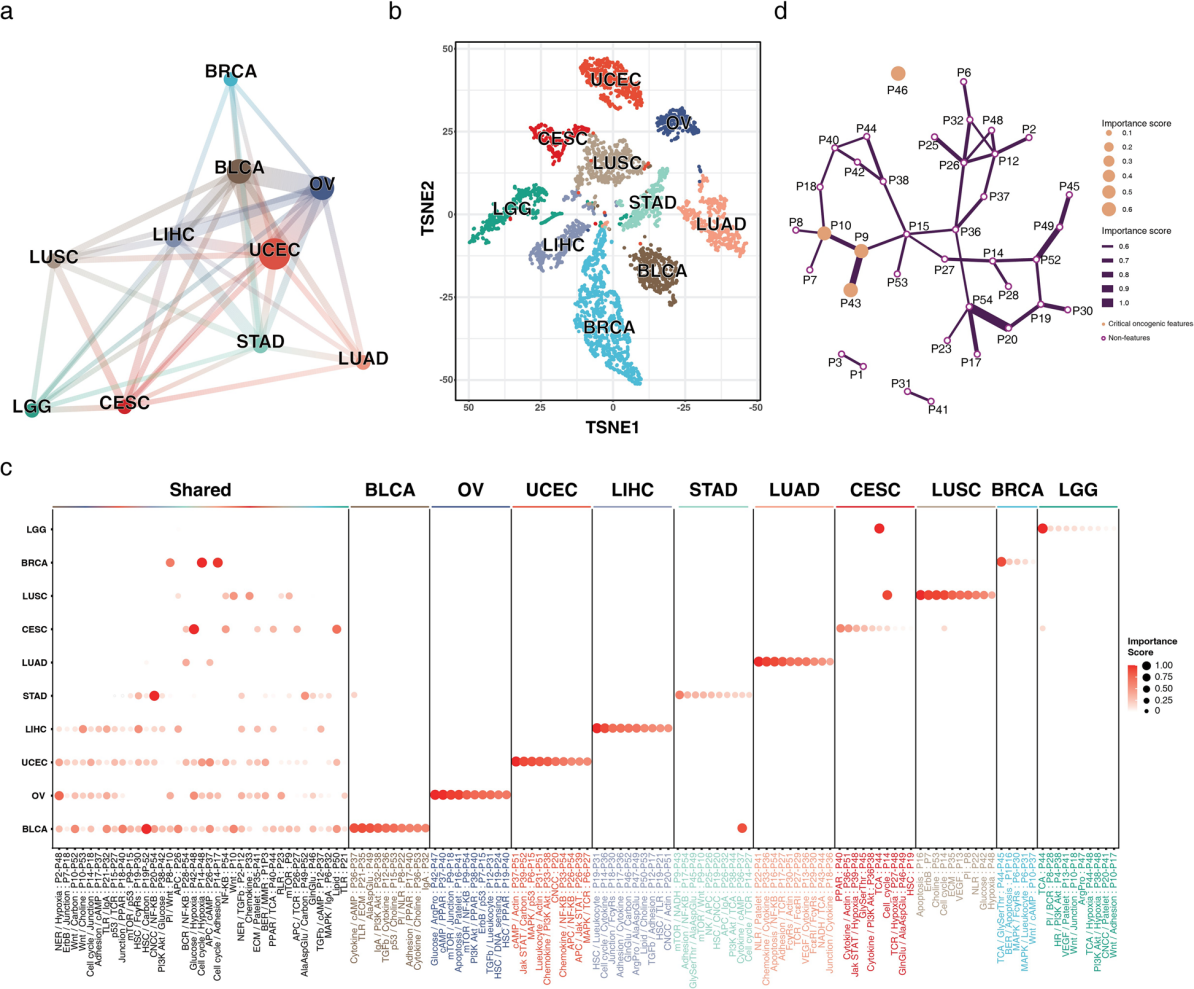
Distribution of critical oncogenic features (COFs) across different cancer types. **a** Shared COF pan-cancer network. Nodes represent cancer types and edge thickness represents the number of shared COFs. Strong connections are visible between bladder cancer (BLCA), ovarian cancer (OV), and uterine corpus endometrial carcinoma (UCEC), and extend to liver hepatocellular carcinoma (LIHC) and stomach adenocarcinoma (STAD). **b** Two-dimensional representation of patients with cancer based on their enrichment status on COFs. Each dot and color represent patients with cancer and the cancer type, respectively. Despite the shared COFs, patients with cancer mostly cluster based on cancer types. **c** Shared and unique COFs identified with NetG2P. The importance rank of each COF is represented by the color and size of the dot. COFs found across four or more cancer types were labeled as “Shared”; all other features not included in this list were sorted by their importance rank and displayed per cancer type. COF names are abbreviated according to Additional file 1: Table S12. A large number of COFs are shared between BLCA, OV, UCEC, LIHC, and STAD. **d** Pathway interaction network of STAD. Nodes represent the oncogenic pathway, and links between pathways are the signaling crosstalk. Node sizes and edges represent the importance rank extracted using explainable artificial intelligence

To compare the similarities and differences between cancer types based on their COFs, we constructed a pan-cancer oncogenic feature network illustrating shared COFs across cancer types (Fig. 3a). BLCA, OV, and UCEC shared the highest number of COFs and formed a core in the network. Specifically, these three cancer types shared 70 common oncogenic features, of which 50 were unique to these cancers (Additional file 1: Table S8). The next highest three-cancer-type connections were BLCA, LIHC, UCEC (11 shared), LIHC, OV, UCEC (10 shared) and BLCA, LIHC, OV, UCEC (7 shared). This suggested that several cancer types commonly activated multiple oncogenic signaling pathways and their crosstalk. BRCA demonstrated a distinct lack of shared COFs with LUSC, LGG, CESC, and LUAD (Fig. 3a), indicating potential cancer-specific mechanisms. To test the ability of COFs to classify distinct groups of patients from various cancer types, we performed a dimension reduction analysis across the 10 cancer types. The results revealed high-level clustering of all 10 cancer types according to the COFs in each patient sample, suggesting that cancer type-specific COFs were apparent for individual cancer types (Fig. 3b).

We further examined the commonly shared COFs across cancer types, which revealed six COFs shared among five cancer types. Each cancer type had at least one of these commonly shared features (Additional file 1: Table S8). These COFs included pathways directly implicated in tumorigenesis (e.g., NF-κB (P54), Wnt (P10), and p53 (P15) signaling), immune-related pathways (e.g., T- and B-cell receptor signaling (P27 and P28), antigen processing and presentation (P26)), metabolism regulation (e.g., adenosine monophosphate metabolism (P37), and carbon metabolism (P52)). Five cancer types (BLCA, OV, UCEC, LIHC, and STAD) formed the shared COF pan-cancer network core (Fig. 3a), which shared COFs involving Wnt signaling, NF-κB signaling, and cell cycle (Fig. 3c).

We also identified 222 cancer-specific COFs, of which ErbB signaling (P7), PI3K-Akt signaling (P38), and actin cytoskeleton regulation (P51) were most frequently connected to other pathways. However, no central pathway within the unique COFs formed numerous links with other pathways, suggesting that each cancer type exhibited distinct patterns of pathway interactions despite sharing some common COFs. To visualize these diverse patterns, we constructed pathway interaction networks for all 10 cancer types. These networks consisted of oncogenic pathways as nodes and signaling crosstalk as links, and revealed diverse COF connectivity patterns (Additional file 2: Figure S1). In many cases, COFs were sufficiently connected to form large clusters, highlighting the complex interplay between oncogenic features in cancer progression.

### Network structural analysis reveals distinct organization patterns of COFs

**Fig. 4:**
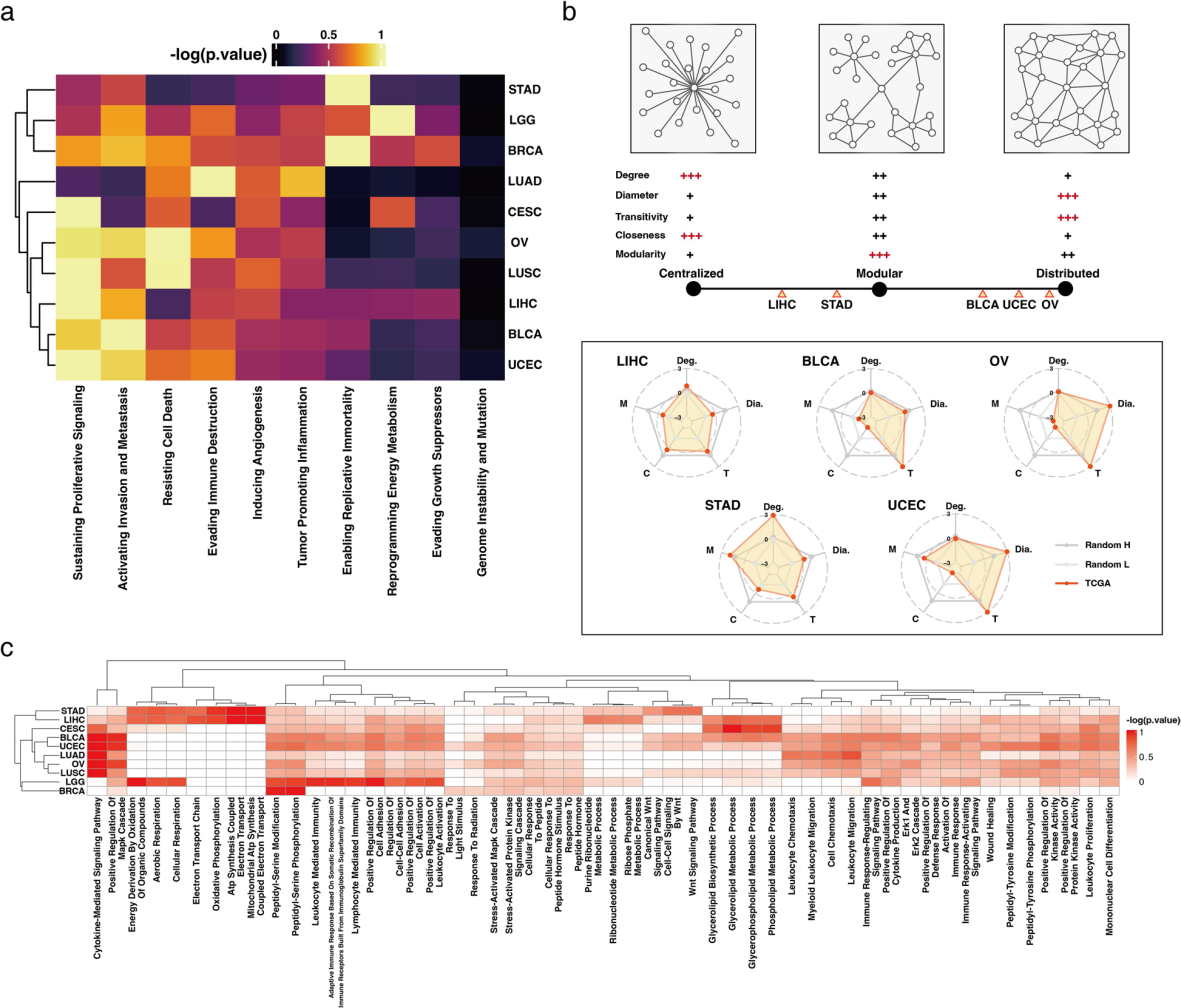
Structural and functional analysis of critical oncogenic features (COFs) and pathway interaction networks. **a** Heatmap of enrichment scores on 10 cancer hallmarks with COFs. Negative log values of *p*-values have been normalized and represented. Higher enrichment is evident in uterine corpus endometrial carcinoma (UCEC), bladder cancer (BLCA), and ovarian cancer (OV) in sustaining proliferative signaling and activating invasion and metastasis, whereas stomach adenocarcinoma (STAD) and liver hepatocellular carcinoma (LIHC) show limited enrichments. **b** Schematic representation of possible network hierarchies. Based on five metrics of network properties (see Methods), centralized, modular, and distributed network structures were proposed. Pathway interaction networks (Fig. 2c) of five cancer types were analyzed. Based on the metrics score, they were overlayed on continuum of network structures (see Methods). LIHC and STAD are located between centralized and modular, whereas BLCA, OV, and UCEC are located near the distributed network hierarchy. **c** Heatmap of Gene Ontology enrichment analysis with COFs. Hierarchical clustering groups STAD and LIHC together, both of which possess the modular network. BLCA, OV, and UCEC show enrichment on various biological functions.

To understand how pathway interconnections affect prognosis, we analyzed the organization of these interactions using pathway interaction networks (Fig. 3d). Based on the five metrics chosen to quantify the structural characteristics of these networks, we hypothesized a continuum of network structural patterns with distinct organizational schemas ranging from centralized (characterized by a central node connecting all others) to distributed (characterized by extensive crosslinks between all network members without discrimination). The modular structure was in between these extremes, in which local assemblies of “modules” were distributed; however, inter-module connections resembled the centralized scheme. To ascertain significance, we compared the metrics of the cancer-type pathway interaction networks against randomly generated networks of the same size (see Methods for details; Fig. 4b).

Our analysis revealed distinct structural patterns in pathway interaction networks across different cancer types. BLCA, OV, and UCEC exhibited characteristics closer to those of a distributed network structure, whereas STAD and LIHC exhibited properties that were more aligned with a modular network structure (Fig. 4b). Specifically, LIHC and STAD demonstrated higher degree scores, with STAD showing the most significant modularity score—hallmarks of centralized and modular networks. In contrast, UCEC and OV displayed high network diameter and transitivity, typical of distributed networks. The cancer types exhibiting distributed structure formed the shared COF network core and had numerous COFs in common (Fig. 3a). This observation raises a question regarding the translation of these structural differences in network organization to functional differences in cancer phenotypes.

Based on network structure, the mutational influences in the distributed network (BLCA, OV, and UCEC) are likely to be shared across many signaling pathways, potentially causing a broader range of biological malfunctions. Conversely, in the modular network (STAD and LIHC), mutational impacts are expected to be localized within modules before spreading to other network modules. These structural tendencies are evident in the enrichment scores of the 10 cancer hallmarks (Fig. 4a). The modular type cancers, STAD and LIHC, show high enrichment in only one hallmark each: enabling replicative immortality and sustaining proliferative signaling, respectively. In contrast, the distributed type cancers, BLCA, OV, and UCEC, exhibit high enrichment across multiple hallmarks, including sustaining proliferative signaling, activating invasion and metastasis, resisting cell death, and evading immune destruction.

Gene ontology (GO) analysis also revealed a similar trend, which grouped cancer types based on their network types (Fig. 4c). STAD and LIHC, the modular type cancers, were uniquely enriched in processes such as the electron transport chain and mitochondrial ATP Synthesis, and also showed strong enrichment in ribonucleotide metabolic process and Wnt signaling. In contrast, the distributed type cancers (BLCA, OV, and UCEC) exhibited a broader range of enrichments, including cytokine-mediated signaling pathway, mitogen-activated protein kinase cascade regulation, peptidyl-serine modification, leukocyte migration, and cell chemotaxis. The modular type cancers demonstrated weaker or no enrichment in the biological functions that were prominent in the distributed type cancers. Overall, the pan-cancer analysis based on COFs revealed distinct patterns of signaling pathway interactions across different cancer types, suggesting that the underlying architecture of pathway interactions may be crucial in determining cancer behavior and potentially developing therapeutic strategies.

### COFs delineate potential drug response biomarkers through perturbation data

**Fig. 5:**
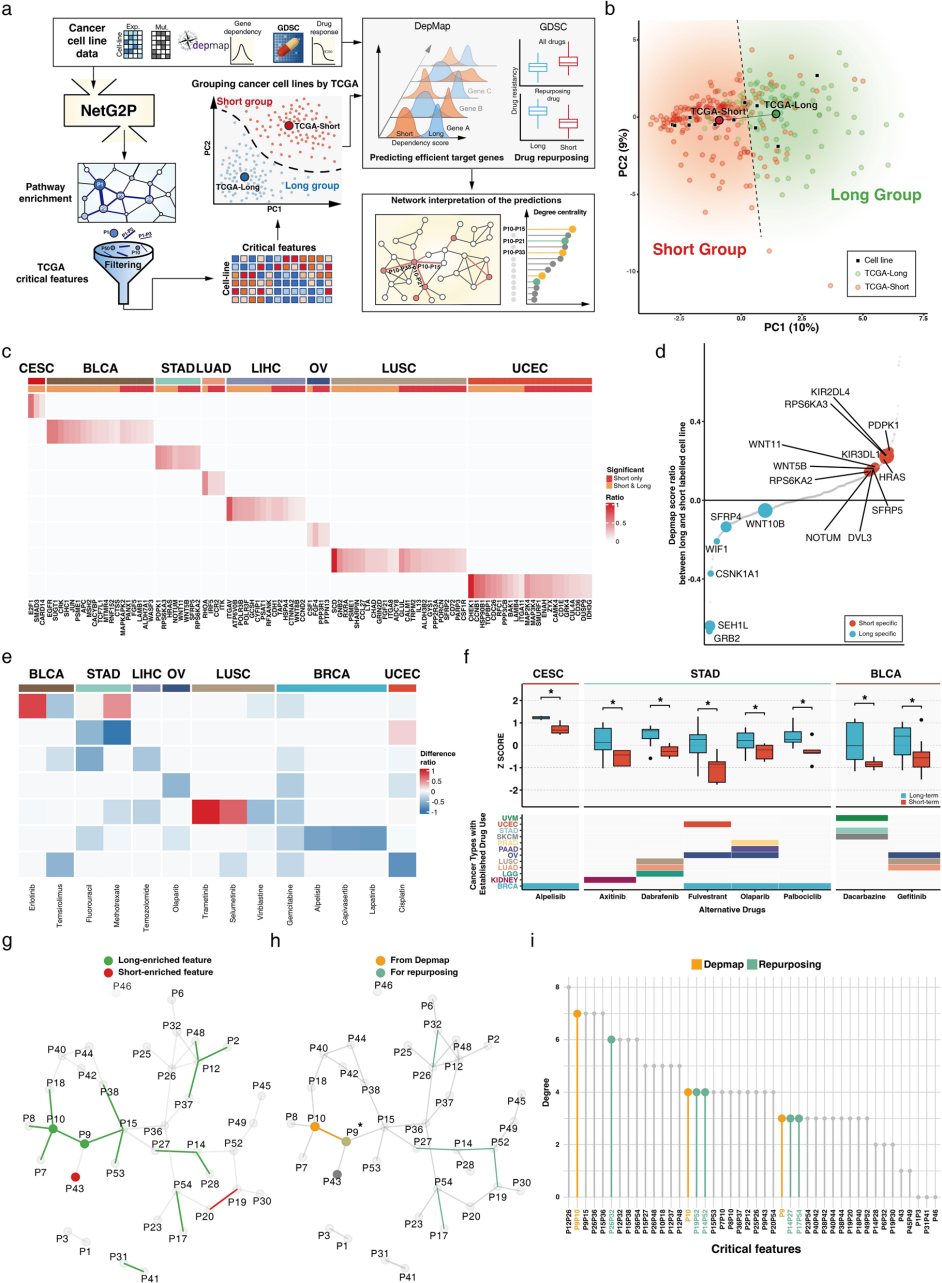
Application of network-based genotype-to-phenotype transformation (NetG2P) and critical oncogenic features (COFs) to identify drug targets and drug repurposing candidates. **a** Workflow integrating cancer cell lines with TCGA data. DepMap provides gene knockout dependency scores, and GDSC provides drug response data. COFs from TCGA filter oncogenic features in cell lines, which are categorized into risk-associated groups via dimension reduction. Differential gene dependencies and drug responses between short- and long-term groups are identified and overlaid on pathway interaction networks. **b** Principal component analysis of STAD patients and cell lines. Cell lines (squares) were classified based on proximity to TCGA group centroids. **c** Differential DepMap scores of genes specific to short-term risk groups. Genes with lower dependency scores in short-term cell lines were identified; “short only” indicates genes with negative scores exclusively in short-term groups. **d** Differential DepMap scores for STAD COFs. Significant genes are colored and labeled; circle size represents negative log *p*-values. **e** Differential drug response between risk groups. Approved or TCGA-used compounds were compared: red indicates effectiveness against short-term, blue against long-term groups. Approved anticancer compounds generally show preferential effectiveness against long-term groups. **f** Drug repurposing candidates for short-term risk groups. Eight approved anticancer compounds showed higher effectiveness against short-term groups of other cancer types. Boxes indicate cancer types for which compounds were originally approved. **g,h** STAD pathway interaction network with COFs, DepMap, and drug repurposing targets. The modular network structure comprises long-term enriched features. Differential survival score genes belong to P9 and P10 pathways; repurposing targets project onto P14-P27 and P17-P54. **i** Structural importance of COFs and potential targets. Features selected via differential response analysis showed higher degrees; DepMap-selected features ranked second

NetG2P identifies critical features of signaling crosstalk that are potentially crucial in cancer prognosis, suggesting that genes within COFs could be drug target candidates for their corresponding cancer types. However, as a large number of COFs were identified in each cancer type, prioritizing and validating optimal drug targets using drug response databases is necessary. Therefore, we used cell line perturbation data to evaluate COFs and the treatment response. If cancer cell lines can capture the tumor phenotypes of short- and long-term risk groups, results from perturbing these cell lines could be extrapolated to understand the prognostic differences in patient groups. Two major perturbation databases utilize cancer cell lines: Genomics of Drug Sensitivity in Cancer (GDSC), which measures cancer cell survival after perturbation with external compounds, and dependency map (DepMap), which measures cell survival after gene knockout using CRISPR technology. These databases were used to assess drug responses between risk groups and identify key genes with COFs that influence the cancer prognosis (Fig. 5a).

We applied the G2P module to cancer cell line genomic data to obtain the oncogenic feature matrix, and then filtered this data using the COFs list derived from TCGA data (see Methods for details). To visualize the relationship between cell lines and patient data, we performed principal component analysis on the combined COF results of cell lines and patient data for corresponding cancer types (Fig. 5b and Additional file 2: Figure S5). We observed that the cancer cell lines were well-distributed around the TCGA short- and long-term risk group centers. Based on their proximity to these centers, we categorized the cell lines into short- and long-risk groups.

DepMap provides dependency scores for each gene, reflecting cell survival rates after gene knockout. We hypothesized that genes within COFs could exhibit different dependency scores across risk groups because of the differential enrichment of biological functions between these groups. To test this hypothesis, we compared dependency scores of short- and long-term risk cell lines. As anticipated, many genes within the COF pathways showed differential dependency scores between these risk groups (Fig. 5c–d and Additional file 2: Figure S6). Overall, we identified 426 genes with significant differences in dependency scores between short- and long-term risk group cell lines (Additional file 11: Table S9). Genes with higher dependency scores in the short-term risk group emerged as promising drug target candidates, which could potentially offer tailored therapeutic strategies for patients requiring rapid intervention.

The genes WNT5B and WNT11, which are part of the COF in STAD (Fig. 5d), demonstrated significantly lower DepMap scores in the short-term risk group. WNT5B and WNT11 are well-established biomarkers of non-canonical Wnt signaling and have been evaluated for their potential use as therapeutic targets in cancers including breast, lung, and intestines cancers [29, 30]. SFRP4 and SFRP5 have previously been investigated on the biphasic behavior of the Wnt pathway [31–33]; they also exhibited higher dependency scores in the short-term risk group.

We hypothesized that drug resistance could be a contributing factor to short-term risk and compared the general tendency of drug resistance via analyzing mean half maximal inhibitory concentration (IC_50_) values. Short-term risk cell lines from seven cancer types showed higher resistance overall (Additional file 2: Figure S7). This trend persisted when we filtered the perturbants to include only compounds used for anticancer treatments in TCGA. Of the 14 compounds identified in TCGA cohorts for treating patients with cancer, only 3 showed higher effectiveness in short-term risk groups (Fig. 5e). Particularly in STAD, two commonly utilized treatments, 5-fluorouracil and methotrexate, were less effective in short-term risk cell lines. Moreover, six compounds demonstrated preferential effectiveness for long-term risk groups in more than two cancer types. Gemcitabine showed lower effectiveness in short-term risk cell lines in 6 of 7 cancer types (Fig. 5e). These results suggest that patients at short-term risk are likely to be more resistant to conventional anticancer treatments, potentially contributing to their poor prognosis.

Confirming poor responses to conventional treatments from short-term risk groups inspired us to identify potentially effective treatments for short-term risk groups. We extended our analysis of the GDSC database to include a broader range of anti-cancer treatment compounds and compared IC_50_ values between the two risk groups (Additional file 1: Table S10). This approach identified eight compounds that showed higher effectiveness in short-term risk cohorts across three cancer types (Fig. 5f). Five of these eight drugs demonstrated effectiveness against STAD short-term risk cell lines. Two of these drugs, Fulvestrant and Olaparib, were already utilized across multiple cancer types (Fulvestrant: BRCA, OV, UCEC; Olaparib: BRCA, OV, PAAD, and PRAD). Additionally, Axitinib, which targeted vascular endothelial growth factor, could potentially modulate Wnt pathways—a component of long-term COFs in STAD [34].

In the previous section, we categorized pathway interaction networks into “distributed” and “modular” types. To determine the relevance between the structural characteristics of these networks and group-specific drug candidate genes identified in DepMap and GDSC, we performed a hypergeometric test to identify network parts responsible for the differential response. The Wnt pathway and its crosstalk with the mTOR pathway (P10 and P9, respectively) were part of the long-term COFs for STAD (Fig. 5g), which was consistent with the observed differential DepMap scores. Genes identified in DepMap for STAD were enriched within signaling crosstalk related to P10 (Wnt pathway) and P9 (mTOR pathway) (Fig. 5h). The P9-P10 signaling crosstalk exhibited the second-highest degree in the pathway interaction network, suggesting a potentially centralized role within the module (Fig. 5i). Target genes for drug repurposing, which were expected to be effective against short-term risk groups, were enriched in several pathway crosstalk including P14-P27, P14-P52, P19-P52, P17-P54, and P26-P32. Among them, P14-P27 and P17-P54 were identified in long-term COFs. The link between the Cell cycle (P14) and T-cell receptor signaling pathway (P27) exhibited the highest edge betweenness centrality and was normalized by its degree in the link graph, reflecting its crucial role in connecting different modules (Additional file 2: Figure S8). These findings indicate that our COF-based stratification reflects meaningful biological differences, helps identify risk group-specific drug targets, and provides insights into potential therapeutic strategies via network structure analysis. Integrating these approaches can potentially facilitate the development of more personalized and effective cancer treatments, improving the precision of cancer therapy.

## Discussion

Our study reveals the integral role of pathway crosstalk in cancer prognosis via developing a framework that stratifies patients with cancer based on COFs, which comprise oncogenic pathways and their crosstalk. This finding was demonstrated by the high predictive power of the G2P module and successful stratification of patients with cancer into short- and long-term risk groups using the COFs.

To our knowledge, this is the first study that utilizes pathway crosstalk to describe cancer phenotypes. We have chosen pathways and their crosstalk instead of genes to cover the biological functions represented by the ensemble of genes working together. Hence, genetic mutation and expression information were integrated and mapped onto these functional units. The high performance of the G2P module using oncogenic features indicates that our network propagation method of genomic information, coupled with pathway and pathway link representation, is relevant in predicting cancer prognosis. To link oncogenic features to the prognostic outcome in the most optimal way, we employed a machine learning model. NetG2P combined biological knowledge-based network propagation and pathway representation with the machine learning to achieve both interpretability and high prediction accuracy for cancer prognosis.

Machine learning models are often criticized for their “black box” nature, which complicates elucidating the mechanism underlying the model making such predictions [35, 36]. To address this issue, our COF module employed XAI to extract important features for patient stratification based on survival analysis, which successfully ranked oncogenic features based on their relative importance in making predictions. Survival analysis then determined the threshold of oncogenic features that were relevant in creating statistically significant prognostic groups.

Various approaches have been developed to define and separate cancer subtypes using surface markers (e.g., ER +, HER +, PR +, and triple negative subtypes of breast cancer) [37], molecular profiles (e.g., consensus molecular subtypes of colorectal cancer) [38], and mutation profiles (e.g., EGFR mutations in non-small cell lung cancer) [39]. The COFs identified in this study are potential novel prognostic markers to stratify patients and identify those who require rapid interventions. The short-term risk groups are shown to be more resistant to standard anticancer treatment, as demonstrated from the cell line perturbation data (Fig. 5e and Additional file 2: Figure S7). To help identify alternative treatments for short-term risk groups, we have generated the list of drugs that are specifically effective for them for each cancer type (Fig. 5f).

Structural differences of the pathway interaction network between cancer types manifested correspondingly in their functional enrichments, such as cancer hallmarks and GO analysis. We initially hypothesized three main categories of network based on five network metrics (Fig. 4b), which helped identify two distinct groups of cancer types with similar network properties. The distributed network type facilitates unrestricted information flow in all directions, whereas the modular network type contains key links that regulate information exchange between specific modules [40, 41]. The modular network structure may offer two distinct approaches for targeted therapies, of which the first involves disrupting a pathway crosstalk that is densely connected with other pathways within a module. For example, the crosstalk between Wnt (P10) and TOR (P9) signaling in the STAD pathway interaction network, which is predicted as a drug target candidate in our study, is connected with many pathways, indicating a high degree in the link graph (Fig. 5g,h). This suggests that it is a key link within a module, and its disruption may significantly impact tumor survival and growth. The second approach is to target a pathway crosstalk that connects modules, whose removal would separate the functional modules. An example is the crosstalk between the cell cycle (P14) and T-cell receptor signaling (P27) pathways in STAD (Fig. 5g and Additional file 2: Figure S8), which is predicted as a drug for repurposing in our study. This crosstalk has the highest edge betweenness centrality score relative to its low degree in the link graph, suggesting that it to be a key link between functional modules.

In our study, we successfully analyzed 10 of 33 cancer types in the TCGA database. The remaining cancer types were excluded because of an insufficient number of patients, or highly skewed ratios of deceased to alive patients. Among the cancer types that met the initial criteria for analysis, COADREAD and kidney cancer could not be analyzed because of statistical insignificance in survival analysis. The inability to identify COFs in colorectal cancer may be attributed to the known association between consensus molecular subtypes and varying prognosis in colorectal cancer [38]. Kidney cancer in our study included KICH, KIRC, and KIRP in TCGA. Separating these subtypes could yield better predictive power, although these individual subtypes did not pass the minimum criteria for NetG2P analysis currently and therefore could not be analyzed.

The primary limitation of our pipeline is the insufficient sample size to effectively train the machine learning module. Hence, only a subset of the TCGA cohorts were analyzed. Future studies and clinical trials with sufficient number of patients would be ideal for training NetG2P on the currently unanalyzed cancer types. Particularly in cases of clinical trials where specific treatment regimen appears to be only affecting a specific group of patients, NetG2P will be able to identify the underlying resistance mechanism at the pathway and pathway links level. NetG2P currently uses an undirected protein–protein interaction network (PPIN) as the propagation backbone. Although directed networks (e.g., gene regulatory networks or curated signaling graphs) can better represent causal signal flow, high-quality direction/sign annotations are often incomplete and context-dependent, and inferred directed networks can vary substantially across methods and tissues. We therefore prioritized an undirected PPIN to maximize coverage, robustness, and reproducibility across cancer types. Incorporating well-validated directed regulatory/signaling networks—where available—may further improve the ability to link mutations to downstream pathway activity and prognosis, and represents an important direction for future work. In summary, our study demonstrates that pathway interactions serve as functional biological units that influence cancer progression and prognosis. Utilizing these pathway interactions as COFs has enabled us to effectively stratify patients with cancer and identified short-term risk groups that require rapid interventions. The pathway interaction network structure provides insight into novel targets for anticancer treatment. Applying our approach to drug response datasets will facilitate the identification of potential candidates for drug repurposing.

## Conclusions

This study presents NetG2P, a network-based framework that translates genomic alterations into pathway crosstalk features to predict cancer prognosis with both high accuracy and interpretability. Pan-cancer analysis revealed distinct structural patterns—distributed versus modular networks—that highlight cancer-type specific mechanisms and therapeutic opportunities. By integrating perturbation data, NetG2P identified risk group–specific drug targets and repurposing candidates, particularly for short-term risk patients resistant to standard therapies. Overall, NetG2P provides a pathway-level perspective that bridges complex mutation patterns with precision oncology applications.

## Methods

### Data processing

TCGA-COAD and TCGA-READ were merged to form COADREAD; TCGA-KICH, TCGA-KIRC, and TCGA-KIRP were merged to form KIDNEY. The cancer types with a patient count of 200 or more, with a vital status ratio between 0.15 and 0.85 were selected (Additional file 2: Figure S9). For survival analysis, “days_to_last_follow_up” for surviving patients and “days_to_death” for the deceased patients was utilized. We selected cohorts with matching cancer types and complete data required for the NetG2P pipeline and further filtered them to have a vital status ratio between 0.15 and 0.85 and a median follow-up > 1 year (Additional file 2: Figure S9). A protein*–*protein interaction network (PPIN) aggregated from five different sources was used as the backbone for network propagation. Only the interactions that appear in at least two of the five databases were kept, and the largest connected cluster of interactions were kept (Additional file 1: Table S11) [42–46](version 11.0). Oncogenic features consisted of 54 oncogenic pathways selected from the Kyoto Encyclopedia of Genes and Genomes database and their crosstalk [47] (Additional file 1: Table S12). Unless otherwise stated, all data processing was performed using R version 4.2.1 or 4.3.1.

### Defining signaling crosstalk

Biologically, we define pathway crosstalk as the shared components between pathways. Operationally in NetG2P, we define pathway crosstalk (“pathway-links”) using KEGG gene-set overlaps:

- Let each KEGG pathway (P_i_) have gene set (G_i_).

- Define crosstalk between pathways (P_i_) and (P_j_) as the overlap gene set

$${C}_{\left\{ij\right\}}= {G}_{i}\cap {G}_{j}$$

- Each non-empty C_{ij}_ becomes a pathway-link feature. Across the curated 54 KEGG pathways, this yields 644 non-empty pathway-link features. These were treated and scored the same way as 54 oncogenic pathways.

### NetG2P construction

NetG2P comprises two modules: The G2P and COF modules (Fig. 1, top). NetG2P requires three types of information: (1) Genetic expression (transcriptome data: bulk RNA-seq), (2) Genetic mutation (SNV data), (3) Clinical data (vital status and duration of patients). All training and validation data were obtained from TCGA and processed in the previous section.

### G2P module

Expression and mutation information was integrated into the data using a network propagation technique. Network propagation was performed with alpha value of 0.7, as described by Shin et al. (2017) [15]. Briefly, mutation effects were estimated through a diffusive process along the PPIN. Therefore, if one gene was mutated, the adjacent genes that interacted with the mutated gene were also affected. Random Degree Preserving Networks (RDPN) were generated based on reference PPIN and the network propagation was repeated. The *p*-value for each gene was calculated based on the proportion wherein the PPIN-generated network propagation value was greater than RDPNs [48]. RDPNs were iterated for 150 times for statistical significance testing. Significantly affected genes (*p* < 0.05) were mapped onto the PPIN, and the patient gene set used for enrichment was defined as the largest connected component (giant cluster) within the PPIN among these significant genes. Genes within the giant cluster were mapped to the oncogenic features using a hypergeometric test, using as background the set of genes for which network propagation *p*-values were available for that patient (i.e., the propagation output/PPIN universe used for testing). Genes within the giant cluster were mapped to the oncogenic features using a hypergeometric test. A matrix of *p*-values for each patient corresponding to each oncogenic feature was transformed with a negative log. This was referred to as the oncogenic feature matrix.

### COF module

Machine learning-based models were trained using an oncogenic feature matrix with clinical data. The data were split into an 8:2 ratio and used to train the Gradient Boosting Machine and Deep Neural Network with fivefold cross-validation (Fig. 2a). The final model was selected based on optimized hyperparameters and cross-validation performances. The models were trained with random grid search parameters using the R package H2O (version: 3.38.0.3, 3.40.0.4, and 3.44.0.3) [49]. From the best-performing model (Additional file 1: Table S13 and S14), the relative prediction relevance was extracted for all oncogenic features using XAI (Fig. 1, top right). For the deep neural network (DNN), we used the Gedeon method [50] to compute feature importance. For the gradient boosting machine (GBM), feature importance was calculated using Friedman’s gradient boosting framework [51]. Oncogenic features were sorted by importance and sequentially added for hierarchical clustering to stratify the patients into two groups. Using the Kaplan–Meier estimator and log-rank test with the R package (survival: 3.5–7, survminer: 0.4.9), the significance between the prognosis of the two groups was measured [52]. The number of oncogenic features was selected based on the lowest *p*-value observed between the two groups, with the minimum group size of 30% of the total patients. Based on the prognostic outcomes, each cohort was classified into either a short- or long-term risk group. Oncogenic features that stratified short- and long-term risk groups were known as critical oncogenic features (COFs).

To calculate associations of COFs and prognostic groups, pairwise *t*-tests were performed between the two risk groups with the oncogenic feature matrix. Any COFs with *p* values > 0.05 were classified as “unassigned,” and the others were classified as short- and long-term-associated based on the mean score of each group.

### Validation

#### Drug-matched survival stratification in TCGA

We extracted drug treatment records standardized drug names using a curated alias mapping (e.g., “5-FU”/“5-Fluorouracil” → “Fluorouracil”). Cancer–drug subsets with sufficient sample size (N ≥ 20 treated patients) were analyzed with NetG2P.

#### Benchmarking against existing network and pathway-based methods

Comprehensive benchmarking was conducted comparing NetG2P against the following methods: HotNet, DawnRank, TieDIE, and PROGENy [12, 53–55]. To ensure a fair comparison, we kept the downstream feature construction (pathway feature generation) and ML pipeline identical wherever possible, and replaced only the upstream step that defines the method-specific per-patient gene sets/representations (NetG2P propagation + giant-cluster extraction) with each alternative method. All network-based methods used the same undirected PPIN backbone, and all methods used the same TCGA mutation data (and expression data where applicable). PROGENy is signature-based and does not require the PPIN; accordingly, we used PROGENy’s native pathway activity scores as features without the enrichment step used for gene-set–based methods (See Additional file 4 for details).

#### External validation

External validation was performed with two independent data sources: LinkedOmics (CPTAC cohorts) [22] and ICGC (International Cancer Genome Consortium; LIRI-JP hepatocellular carcinoma cohort accessed via UCSC Xena) [23]. We selected cohorts with matching cancer types and complete data required for the NetG2P pipeline, and further filtered them to have a vital status ratio between 0.15 and 0.85 and a median follow-up > 1 year (Additional file 1: Table S5). This yielded three datasets: CPTAC-LSCC, CPTAC-LUAD, and ICGC-LIHC (LIRI-JP). These were analyzed using the same NetG2P feature-construction pipeline to generate the per-patient pathway and pathway-link feature matrix. We filtered the external feature matrix to the pre-defined COF set identified from the corresponding TCGA cancer type, thereby obtaining an external COF matrix for downstream risk stratification.

### Pan-cancer representation and dimension reduction analysis

COFs from each cancer type were represented as a pan-cancer network, in which each node, its size, and its edge represented a cancer type, the log-transformed number of COFs for the cancer type, and the number of shared COFs, respectively. Dimension reduction was performed on the combined COF matrix.

For cancer cell line data and external cohort data, COFs from the respective TCGA cancer type were used to create a subset of the oncogenic feature matrix. Principal component analysis was then performed with the respective patients with the TCGA cancer type. The mean distance between the principal components of patients from TCGA that had a short- and long-term risk was calculated to identify the centroid of each group. The cell lines were then annotated based on their relative distance between the two risk group centroids.

### Pathway interaction network structure analysis

COFs were represented as a pathway interaction network, in which vertices corresponded to oncogenic pathways and edges represented signaling crosstalk between the pathways (Fig. 3d and Additional file 2: Figure S1). To investigate COF network formation and their variations across cancer types, five metrics were introduced to categorize each pathway interaction network as centralized, modular, or distributed (Fig. 4b): degree, diameter, transitivity, closeness, and modularity. The degree metric assessed degree centrality via evaluating the adherence of the degree distribution of the critical feature network with a power law (${x}^{\alpha }$, where $x$ is the network degree and $\alpha$ is the power law exponent). In many real-world networks, alpha typically takes values between 2 and 3 [56]. The *p*-value of the Kolmogorov–Smirnov test was used to determine the distribution fitting with a power law; a *p*-value > 0.05 indicated a power law fit, which was the case for STAD and LIHC (Additional file 2: Figure S10). The diameter metric measured the length of the longest geodesic in the network. The diameter was normalized by the total number of edges. Transitivity quantified the probability that the adjacent vertices of a vertex were connected. Closeness centrality was calculated to determine the steps required to access every other vertex from a given vertex. Centralized networks tended to have higher values for closeness centrality. To assess modularity, the network was divided into modules based on the edge betweenness score and the modularity was computed using the membership. The “fit_power_law,” “diameter,” “transitivity,” “closeness,” and “modularity” functions from the igraph library in the R package were used.

### Enrichment analysis

All genes belonging to COFs for each cancer type were used to perform the enrichment analysis. The cancer hallmark enrichment was performed against the gene list of each cancer hallmark [57]. The maximum value negative log of *p*-values was used to normalize the matrix. For GO enrichments analysis, the database “biological function” was selected using the R package CompareCluster (version 4.8.2). The terms were filtered by *q*-value < 0.0001 and the top 15 terms were selected. The negative log of *q*-value matrix was normalized using the minimum–maximum normalization method.

### Total screening of DepMap database

For each gene, the differential dependency score between short- and long-term risk groups was assessed using Student’s *t* test. Any genes with *p*-value < 0.03 were selected as differential dependency genes. The dependency score difference was plotted, with the size of dot representing the negative log *p*-value. The genes were further categorized based on their group mean dependency score; an average score below zero for both groups were indicated as ‘Short & Long,’ whereas when only that of the short-term group was below zero, the score was referred to as ‘Short only.’

### Total screening of GDSC database and drug repurposing analysis

The names of compounds utilized in TCGA clinical data were standardized based on Genomics of Drug Sensitivity in Cancer (GDSC) nomenclature. The approved list of cancer treatment compounds was obtained from NIH cancer treatment (https://www.cancer.gov/about-cancer/treatment/drugs/cancer-type). The *Z*-score normalized IC_50_ was used to assess treatment responsiveness for each prognosis group per cancer type using a two-sample *t*-test. For each cancer type, the approved list of cancer treatments and compounds utilized in TCGA trials were identified and their response was compared between short- and long-term risk groups. For repurposing candidates, cancer treatment options from all other cancer types were screened against the selected cancer type; compounds that showed a stronger effect on short-term risk groups were identified.

## Supplementary Information

Additional file 1. Tables S1-S8, S10-S14. Table S1. The list of Critical Oncogenic Features. Critical oncogenic features for each cancer types identified through NetG2P and their relative importance score from XAI. The features with two Ps indicate pathway interactions between the two pathways, and one P indicates the pathway itself as an COF. Table S2. The MCC value of cancer prediction models for each cancer types The NetG2P ablation study performance measured by MCC. See Figure 2 and methods for details. Table S3. Benchmark scores against other network and pathway-based methods in predicting patients’ vital status. The NetG2P is benchmarked against other network- and pathway-based methods. See Additional file 4 for detailed methods. Table S4. Kaplan-Meier analysis on within the same drug-treated subsets. The TCGA cohorts were filtered to only contain the same cancer-drug treatment pairs. The survival analysis was performed again with remaining patients. Table S5. External samples collected for NetG2P validation. External dataset was collected from LinkedOmics and ICGC, and evaluated whether data met the requirement for NetG2P analysis. Table S6. The number of COFs and their resulting p-value between the risk-associated groups. The statistical significance of short- and long-term risk groups and the COFs that clustered the two groups. Table S7. COFs and their signaling crosstalk. The number of pathways and pathway links each oncogenic features formed. Global.path.link indicates the number of incidences the oncogenic feature appeared as a COF, as itself or part of the signal crosstalk, and the Global.link only the signal crosstalk. The number of interactions is normalized by the size of the pathway to create Global.link.n, and the aggregate rank combining the number of Global.link and Global.link.n was used to score the number of crosstalk. Table S8. COFs across cancer types. Binary table of COFs distribution across cancer types. Table S10. Drug repositioning candidates. For each cancer types, we identified compounds that show statistically significant differences in its effect on short- or long-term risk groups. See methods for statistical evaluation. Table S11. The Protein-protein interaction network used in this study. PPIN was generated as described in methods. Each line indicates the link between the two proteins. Table S12. Oncogenic feature pathways. The pathways important in tumorigenesis obtained from Kegg database, as described in methods. The numbers indicate the internal reference to the pathways. Short and shorter names were used for conciseness in figure captions. Table S13. DNN Model Parameters. Complete model hyperparameters for all cancer types with DNN as the best model. "-" indicates the parameter was not specified and used H2O default values. All models used seed=1 for reproducibility. Table S14. GBM Model Parameters. Complete model hyperparameters for all cancer types with GBM as the best model. "-" indicates the parameter was not specified and used H2O default values. All models used seed=1 for reproducibility.

Additional file 2. Figure S1-S10. Figure S1. Pathway interaction network of 10 cancer types. COFs identified from NetG2P for each cancer types were used to construct cancer-specific pathway interaction network. The node indicates an oncogenic pathway, and link between nodes the crosstalk between the two pathways. Filled nodes indicates that the pathways themselves were also identified as COF, while the empty nodes were not. Figure S2. Comparison of machine learning performance across different methods. Network- and pathway-based methods were used to predict patients’ vital status. Performance for each cancer type was evaluated usingF1 score,Matthews correlation coefficient, andaccuracy. Across all evaluated metrics and cancer types, NetG2P consistently outperformed the other methods.Statistical significance of performance differences was assessed using the Wilcox-on test, demonstrating that NetG2P achieved significantly higher performance than the compared algorithms. Figure S3. NetG2P retains prognostic power within drug-matched patient subsets. Kaplan–Meier survival analyses were performed on eight drug-matched subsets across five cancer types. NetG2P successfully stratified patients into short- and long-term cohorts with statistically significant differences in survival. Figure S4. NetG2P accurately stratifies cancer cohorts from independent external datasets.Kaplan–Meier survival analyses for ICGC-LIHC, CPTAC-LSCC, and CPTAC-LUAD cohorts. Cancer-specific oncogenic factorsderived from TCGA were applied to stratify patients in each external dataset. Patients classified into the short-term risk groupexhibit significantly worse prognosis in two of the three external cohorts.Heatmaps showing COF enrichment scores across patients in the LIRI-JP, CPTAC-LSCC, and CPTAC-LUADcohorts. COFs are grouped by type, and patients are ordered by risk group assignment. Warmer colors indicate higher enrichment scores. Figure S5. Categorization of cancer cell lines into prognosis-related groups. Cancer patients’ data overlaid with their respective cancer type cell lines. The genomic information of the cancer cell lines was integrated as described in G2P module to generate oncogenic feature matrix. Then, COFs from the respective TCGA cancer type were used to create a subset of the oncogenic feature matrix. This data was appended to the oncogenic feature matrix from TCGA patients to perform dimension reduction. Cancer cell lines are well-integrated within the patient’s data. From this map, the cell lines were categorized into their respective groups based on their proximity to the center point of each prognosis groups. Figure S6. Differential DepMap scores in different cancer types. All genes from COFs of each cancer types were analyzed, and differences between short- and long-term risk groups were plotted. Statistically significant genes were colored and labeled; circle size represents the negative log of p-values. Figure S7. General responsiveness of each prognosis-related groups of each cancer types. The normalized z-score of all compounds were compared between short- and long-term risk groups of 9 cancer types cell lines. In LUAD and BLCA, the long-term risk groups showed higher resistance to perturbation with compounds, while in other cancer types the short-term risk groups were more resilient to compounds. Figure S8. Network structure properties of pathway crosstalks in STAD pathway interaction network. For each node in the pathway interaction network of STAD, the edge betweenness was calculated. Edge betweenness is defined as the number of shortest paths passing through the given edge. Since this measurement is naturally skewed towards edges with high connections, it was normalized with the degree of the given edge. Figure S9. Distribution of number of patients and the vital status ratio of TCGA cancer types. The number and the dead/alive ratio of patients with cancer were both considered to select cancer types for NetG2P training and analysis. Only cancer types with patients count of 200 or more with a vital status ratio between 0.15 and 0.85 were selected. For cancer types the TCGA abbreviation was used except for COADREAD, which combined COAD and READ, and KIDNEY, which consisted KICH, KIRC and KIRP. Figure S10. The node degree distribution of pathway interaction networks. The pathway interaction networks constructed with COFs were analyzed for structural properties. The nodes with link number greater than zero were counted and the linear fit was performed. The p-value of the Kolmogorov-Smirnov test was used to determine the distribution fitting with a power law; a p-value > 0.05 indicated a power law fit, which was the case for STAD and LIHC. The alpha values of STAD and LIHC lay between 2 and 3.

Additional file 3. Table S9. For each cancer types, genes within COFs for the cancer type were tested for differential DepMap scores between short- and long-term cell line groups. See methods for statistical evaluation.

Additional file 4. Supplementary methods. NetG2P was compared with other network- and pathway-based methods to compare its performance in predicting vital status of cancer patients as well as predicting prognosis. The file describes detailed procedure for benchmarking configuration.

## Data Availability

The latest TCGA iteration of the transcriptome, mutation, and clinical data was downloaded directly using an application programming interface (TCGAbiolinks: version 2.30.0) via R. The DepMap database (RRID:SCR_017655) was downloaded directly from the website (https://depmap.org/portal/) [58]. The GDSC database was downloaded directly from the website (https://www.cancerrxgene.org/) [59]. The dataset(s) supporting the conclusions of this article including NetG2P model, data preprocessing for network propagation, and COF for predicting are available in the GitHub repository, https://github.com/4to1stfloor/NetG2P and have been archived in the Zenodo repository [60]. All data generated or analysed during this study are included in this published article, its supplementary information files and publicly available repositories.

## Abbreviations

BLCA: Bladder cancer
COF: Critical oncogenic feature
DepMap: Dependency map
GDSC: Genomics of Drug Sensitivity in Cancer
GO: Gene Ontology
IC_50_: Half maximal inhibitory concentration
LIHC: Liver hepatocellular carcinoma
MCC: Matthews correlation coefficient
NetG2P: Network-based Genotype-to-Phenotype Transformation
OV: Ovarian cancer
PPIN: Protein-Protein Interaction Network
RDPN: Random degree-preserving network
SNV: Single nucleotide variant
STAD: Stomach adenocarcinoma
TCGA: The Cancer Genome Atlas
UCEC: Uterine corpus endometrial carcinoma
XAI: Explainable Artificial intelligence
