# Supplementary material for: NetG2P: Network-based genotype-to-phenotype transformation identifies key signaling crosstalk for prognosis in pan-cancer study
Source: BMC Biol. 2026 Feb 24;24:85. doi: 10.1186/s12915-026-02559-x (PMC13037077; doi:10.1186/s12915-026-02559-x)
Supplement: Supplementary file 2 — Additional file 2. Figure S1-S10. Figure S1. Pathway interaction network of 10 cancer types. COFs identified from NetG2P for each cancer types were used to construct cancer-specific pathway interaction network. The node indicates an oncogenic pathway, and link between nodes the crosstalk between the two pathways. Filled nodes indicates that the pathways themselves were also identified as COF, while the empty nodes were not. Figure S2. Comparison of machine learning performance across different methods. Network- and pathway-based methods were used to predict patients’ vital status. Performance for each cancer type was evaluated usingF1 score,Matthews correlation coefficient, andaccuracy. Across all evaluated metrics and cancer types, NetG2P consistently outperformed the other methods.Statistical significance of performance differences was assessed using the Wilcox-on test, demonstrating that NetG2P achieved significantly higher performance than the compared algorithms. Figure S3. NetG2P retains prognostic power within drug-matched patient subsets. Kaplan–Meier survival analyses were performed on eight drug-matched subsets across five cancer types. NetG2P successfully stratified patients into short- and long-term cohorts with statistically significant differences in survival. Figure S4. NetG2P accurately stratifies cancer cohorts from independent external datasets.Kaplan–Meier survival analyses for ICGC-LIHC, CPTAC-LSCC, and CPTAC-LUAD cohorts. Cancer-specific oncogenic factorsderived from TCGA were applied to stratify patients in each external dataset. Patients classified into the short-term risk groupexhibit significantly worse prognosis in two of the three external cohorts.Heatmaps showing COF enrichment scores across patients in the LIRI-JP, CPTAC-LSCC, and CPTAC-LUADcohorts. COFs are grouped by type, and patients are ordered by risk group assignment. Warmer colors indicate higher enrichment scores. Figure S5. Categorization of cancer cell lines into prognosis-rel [file 12915_2026_2559_MOESM2_ESM.docx]

**Supplementary Figure****s**
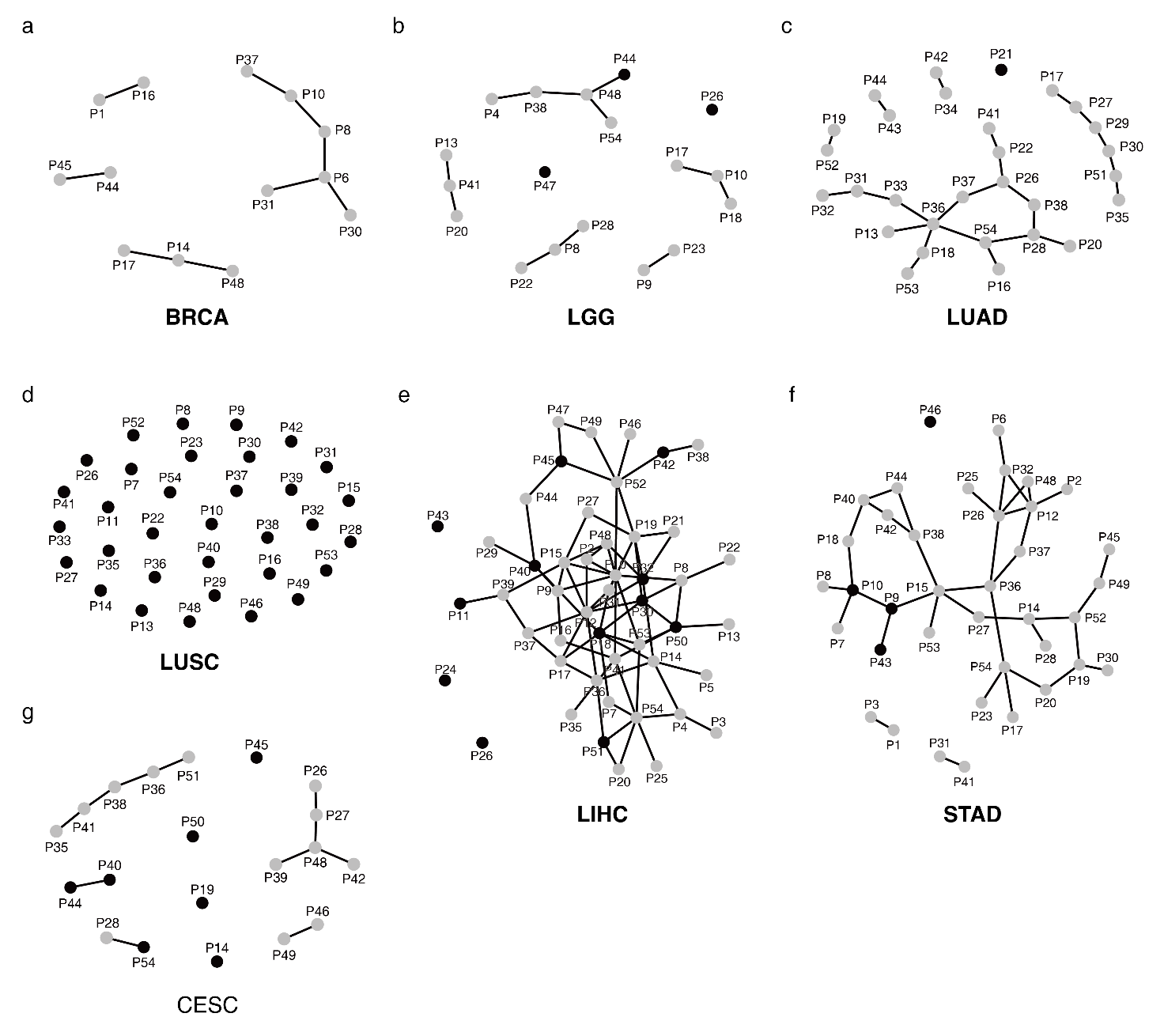

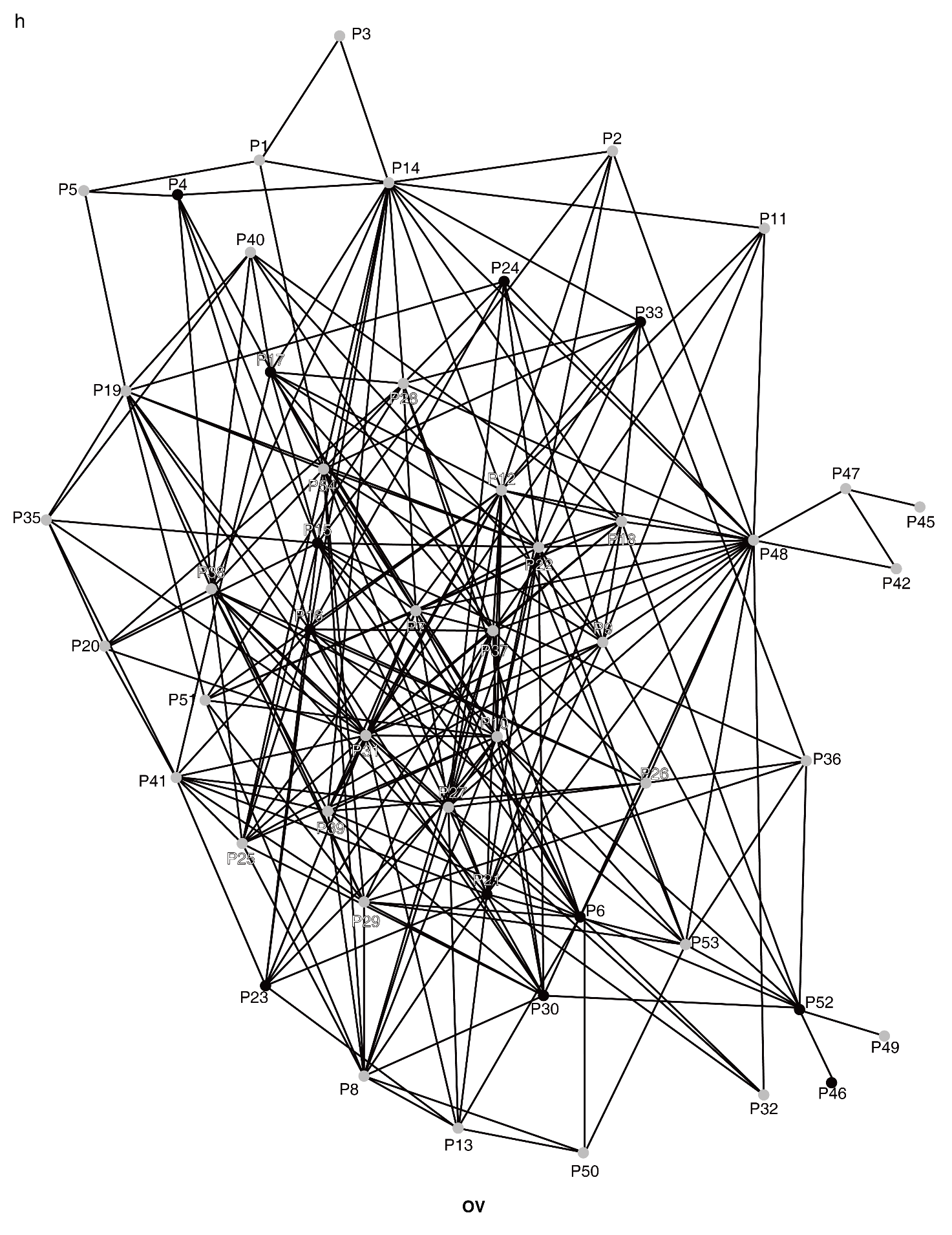


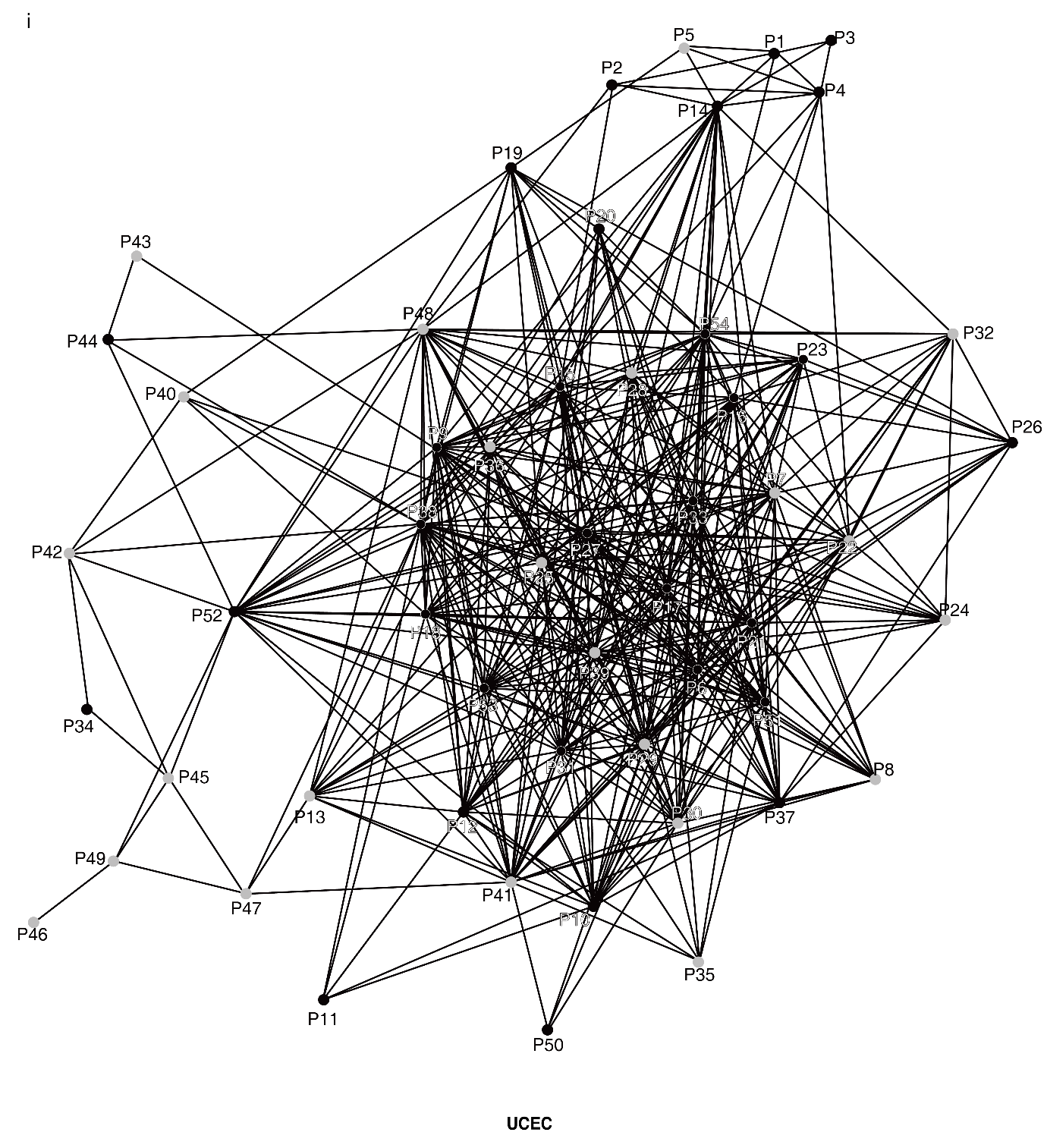


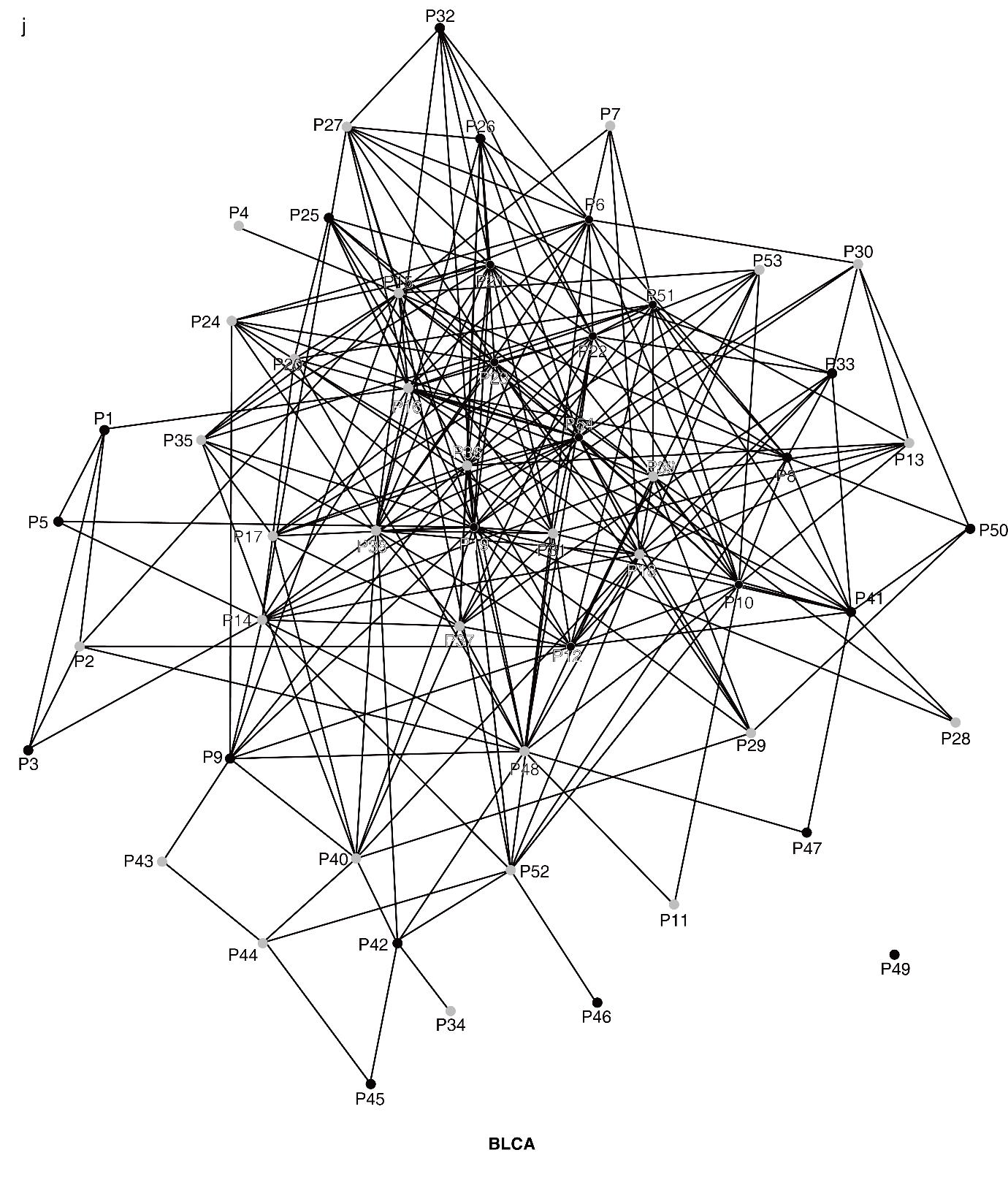


**Figure S1. Pathway interaction network of 10 cancer types**

COFs identified from NetG2P for each cancer types were used to construct cancer-specific pathway interaction network. The node indicates an oncogenic pathway, and link between nodes the crosstalk between the two pathways. Filled nodes indicates that the pathways themselves were also identified as COF, while the empty nodes were not. COF networks of BRCA, LGG, LUAD, LUSC, LIHC, STAD, CESC, OV, UCEC and BRCA (A-J) are shown. LUSC (D) did not show any significant interactions. BRCA, LGG, LUAD and CESC (A, B, C, G) formed locally connected networks. LIHC, STAD, OV, UCEC and BLCA (E, F, H, I, J) formed almost fully connected dense networks.


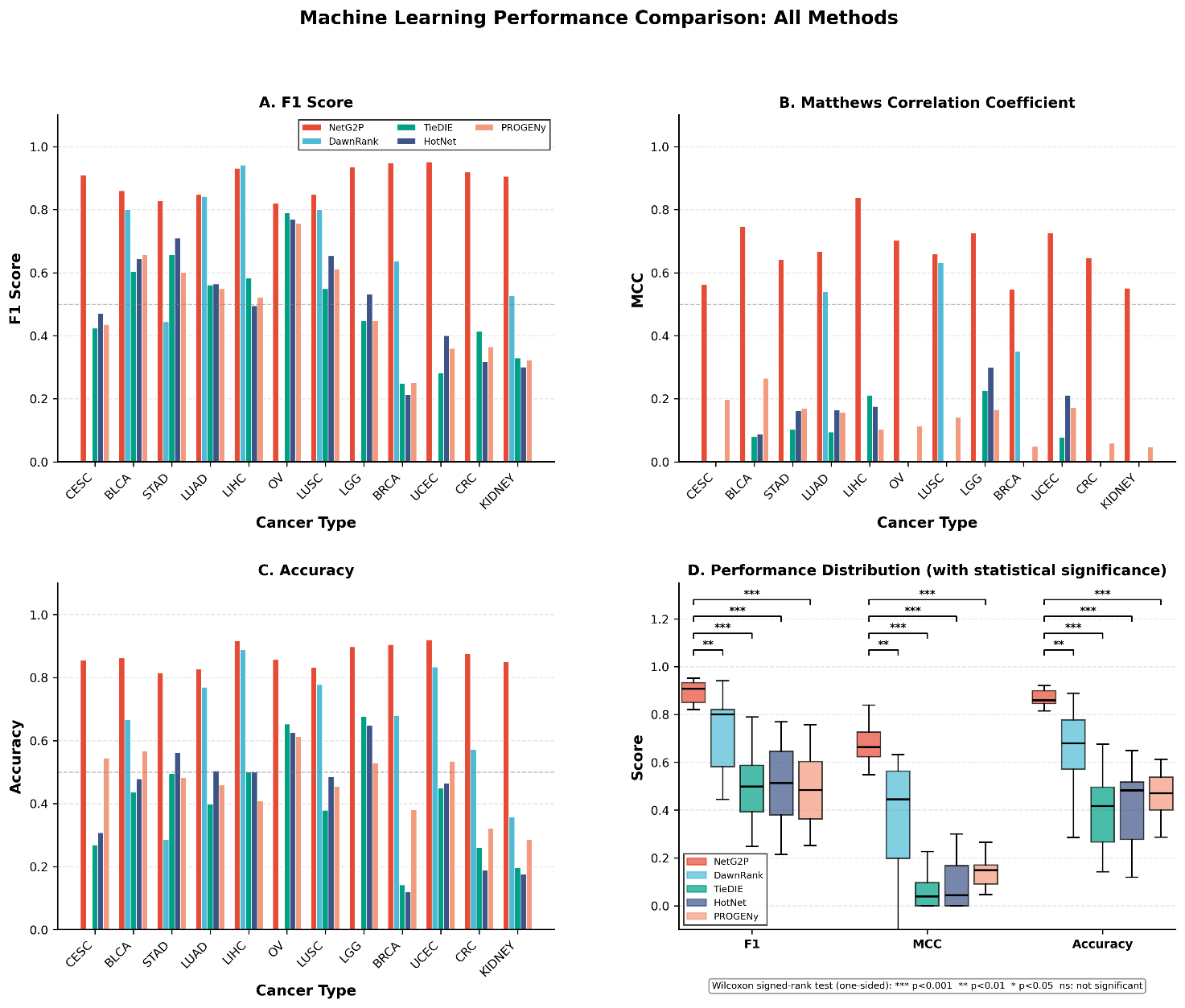


**Figure S2. Comparison of machine learning performance across different methods**

Network- and pathway-based methods were used to predict patients’ vital status. Performance for each cancer type was evaluated using (A) F1 score, (B) Matthews correlation coefficient (MCC), and (C) accuracy. Across all evaluated metrics and cancer types, NetG2P consistently outperformed the other methods. (D) Statistical significance of performance differences was assessed using the Wilcoxon test, demonstrating that NetG2P achieved significantly higher performance than the compared algorithms.


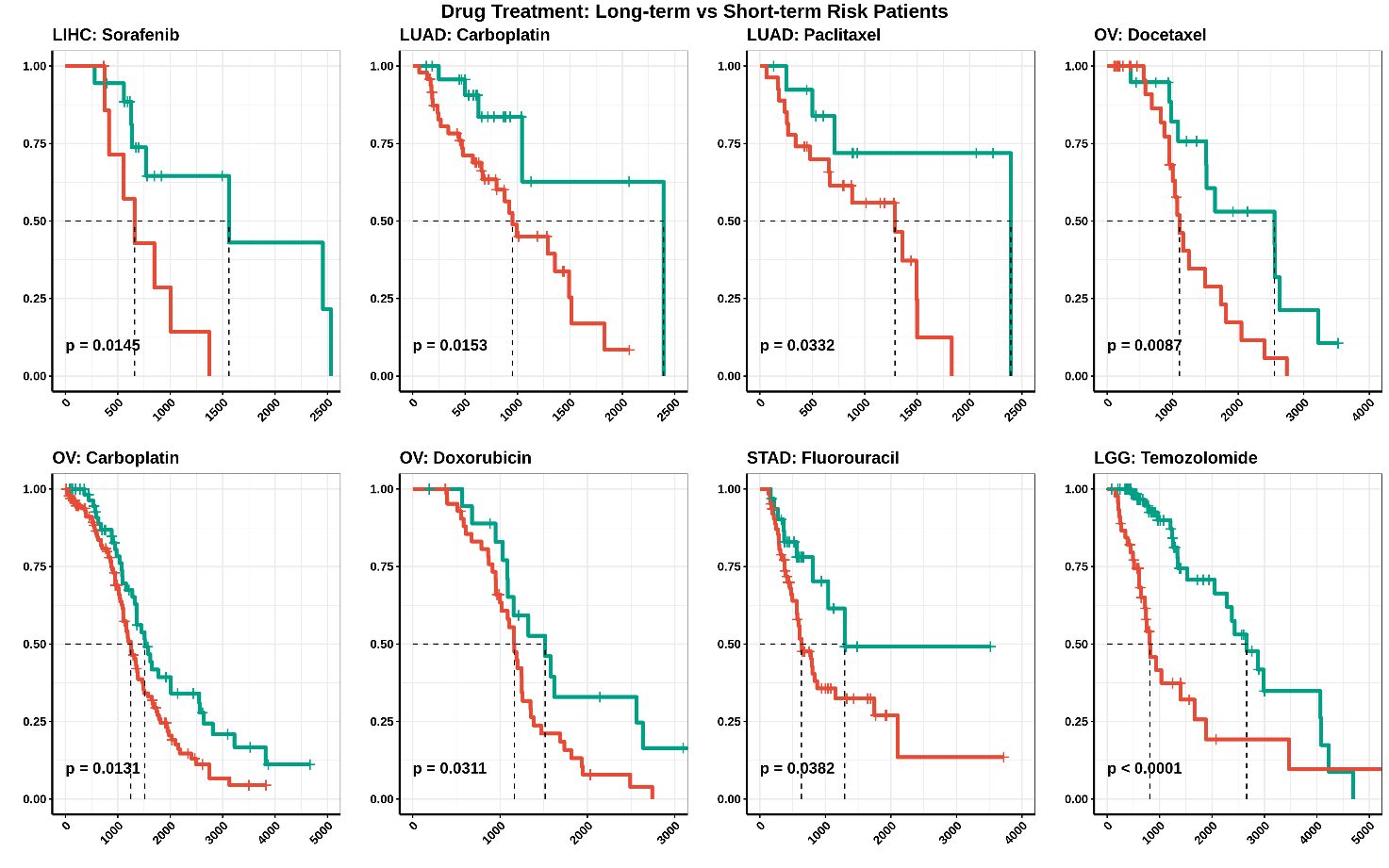
 **Figure S3. NetG2P retains prognostic power within drug-matched patient subsets**

Kaplan–Meier survival analyses were performed on eight drug-matched subsets across five cancer types. NetG2P successfully stratified patients into short- and long-term cohorts with statistically significant differences in survival.


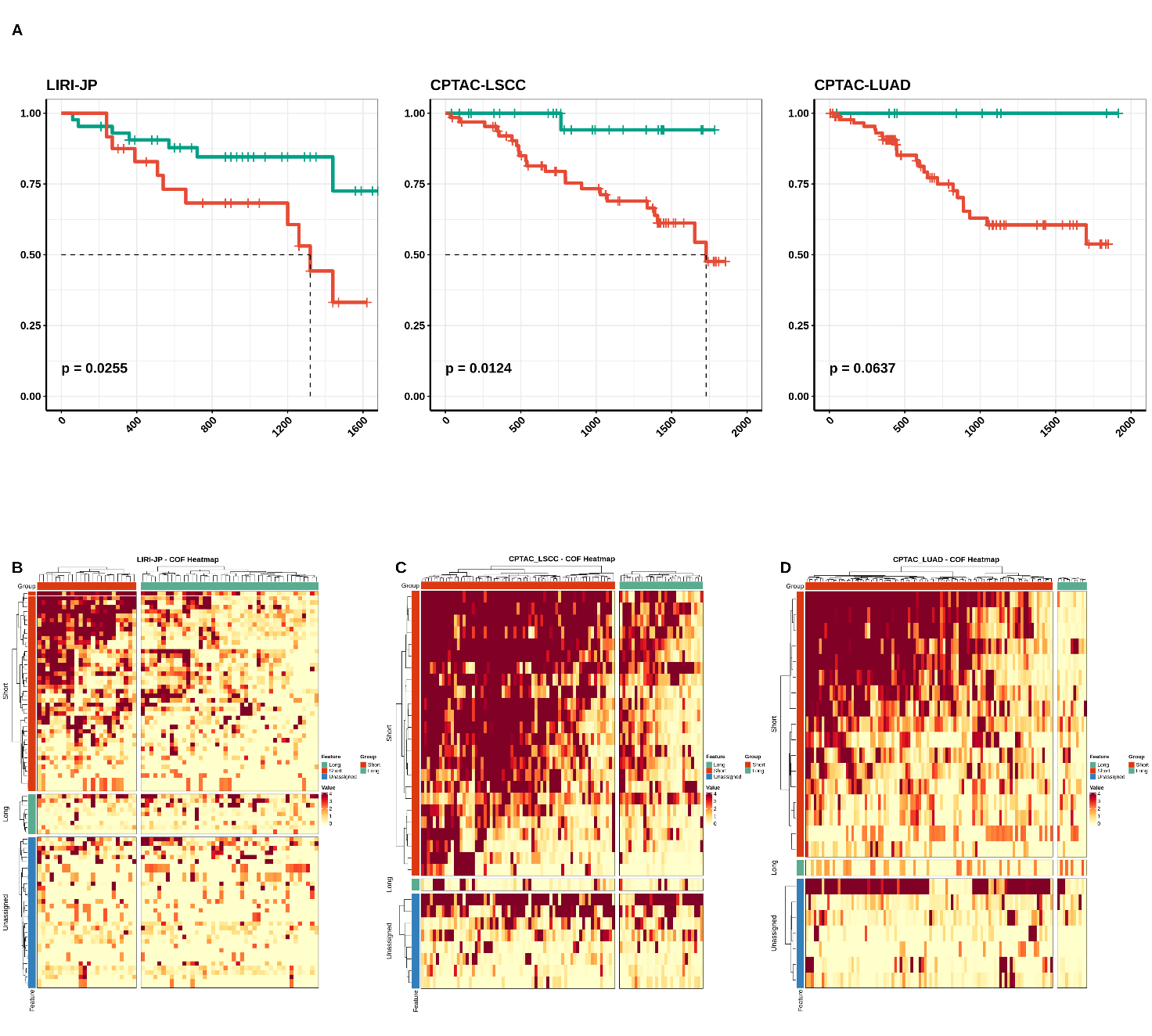
 **Figure S4. NetG2P accurately stratifies cancer cohorts from independent external datasets.**

(A) Kaplan–Meier survival analyses for ICGC-LIHC (LIRI-JP), CPTAC-LSCC, and CPTAC-LUAD cohorts. Cancer-specific oncogenic factors (COFs) derived from TCGA were applied to stratify patients in each external dataset. Patients classified into the short-term risk group (red) exhibit significantly worse prognosis in two of the three external cohorts.

(B–D) Heatmaps showing COF enrichment scores across patients in the LIRI-JP (B), CPTAC-LSCC (C), and CPTAC-LUAD (D) cohorts. COFs are grouped by type (short, long, and unsigned), and patients are ordered by risk group assignment. Warmer colors indicate higher enrichment scores.


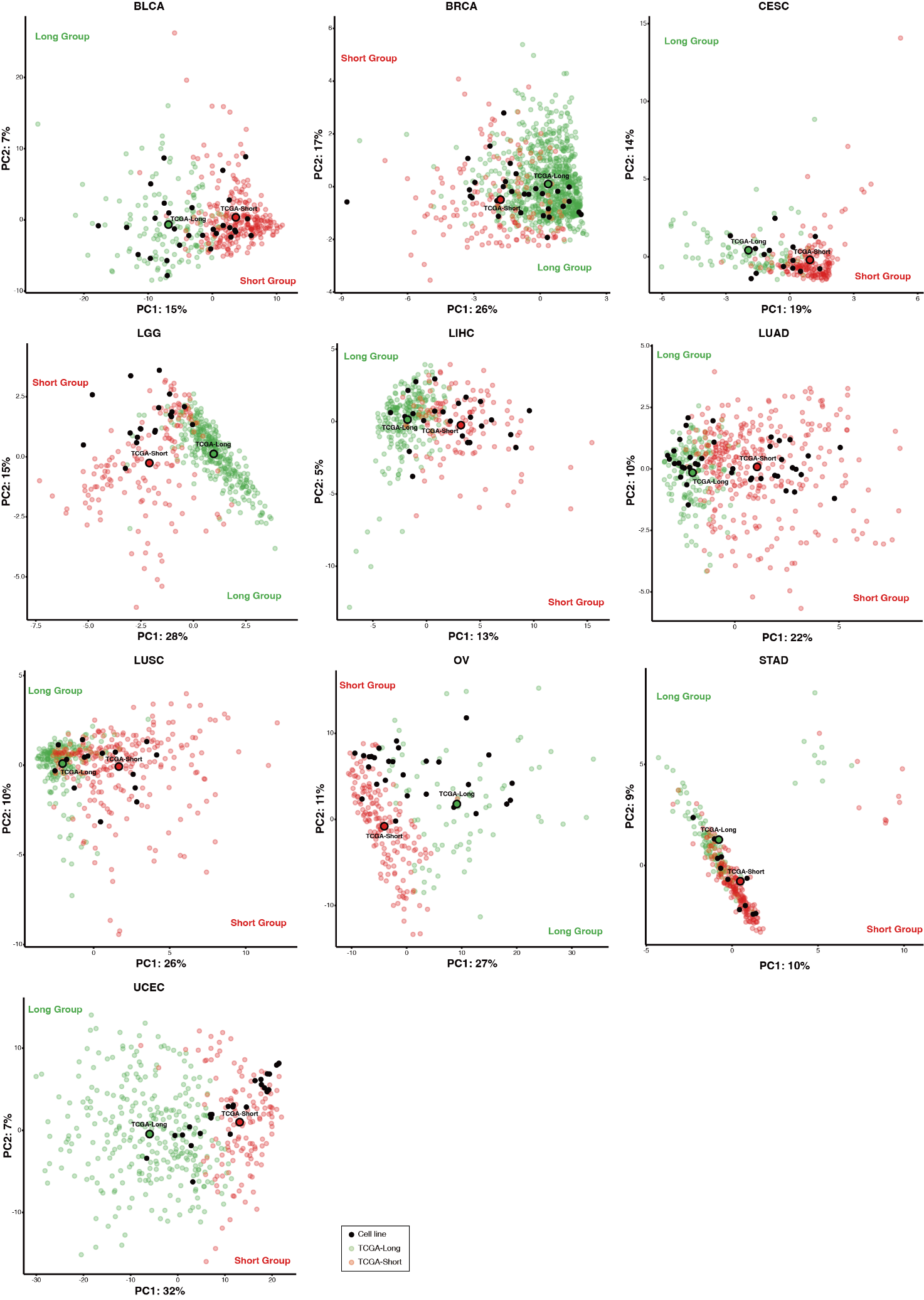


**Figure S5. Categorization of cancer cell lines into prognosis-related groups**

Cancer patients’ data overlaid with their respective cancer type cell lines. The genomic information of the cancer cell lines was integrated as described in G2P module to generate oncogenic feature matrix. Then, COFs from the respective TCGA cancer type were used to create a subset of the oncogenic feature matrix. This data was appended to the oncogenic feature matrix from TCGA patients to perform dimension reduction. Cancer cell lines are well-integrated within the patient’s data. From this map, the cell lines were categorized into their respective groups based on their proximity to the center point of each prognosis groups.


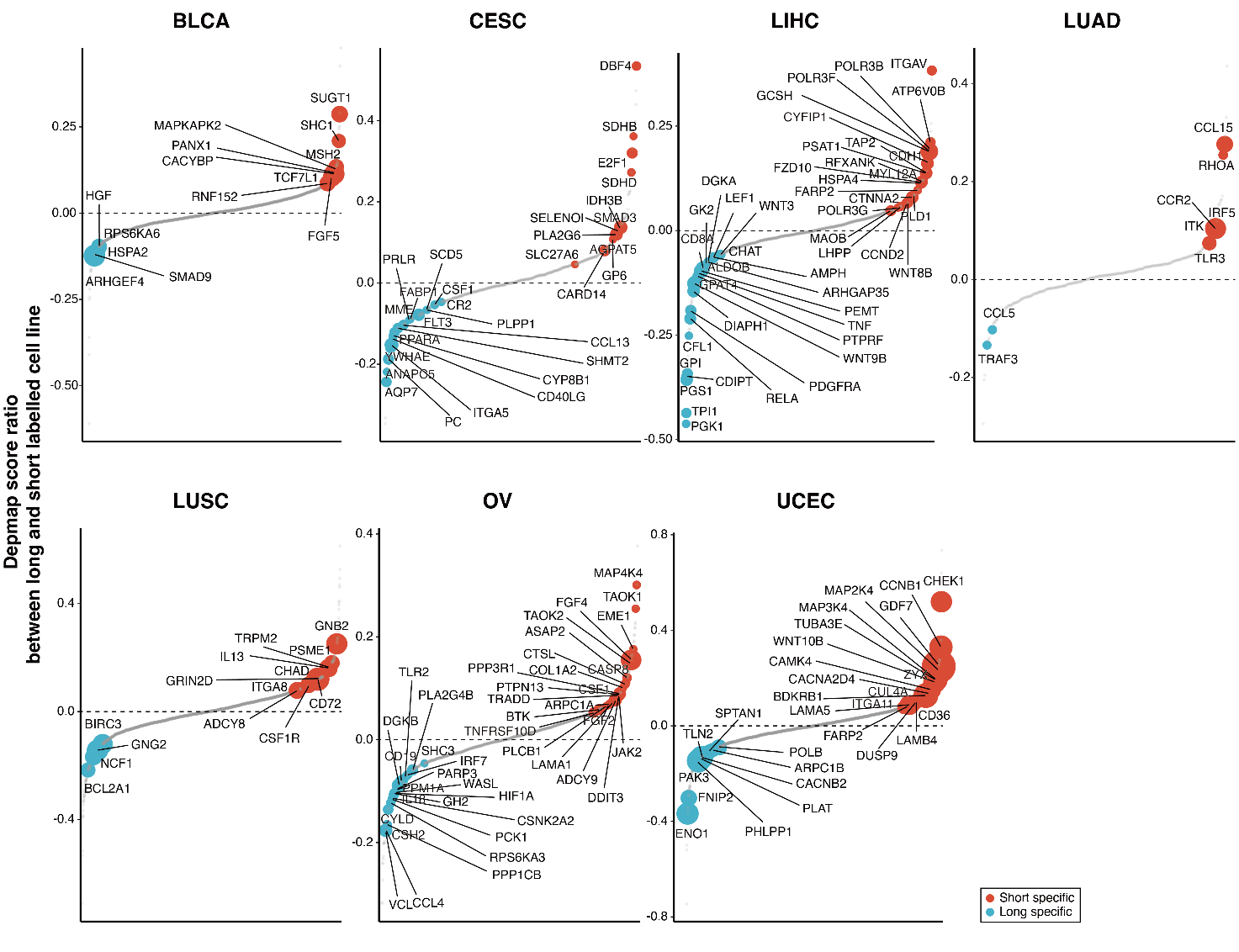


**Figure S6. Differential DepMap scores in different cancer types**

All genes from COFs of each cancer types were analyzed, and differences between short- and long-term risk groups were plotted. Statistically significant genes were colored and labeled; circle size represents the negative log of p-values.


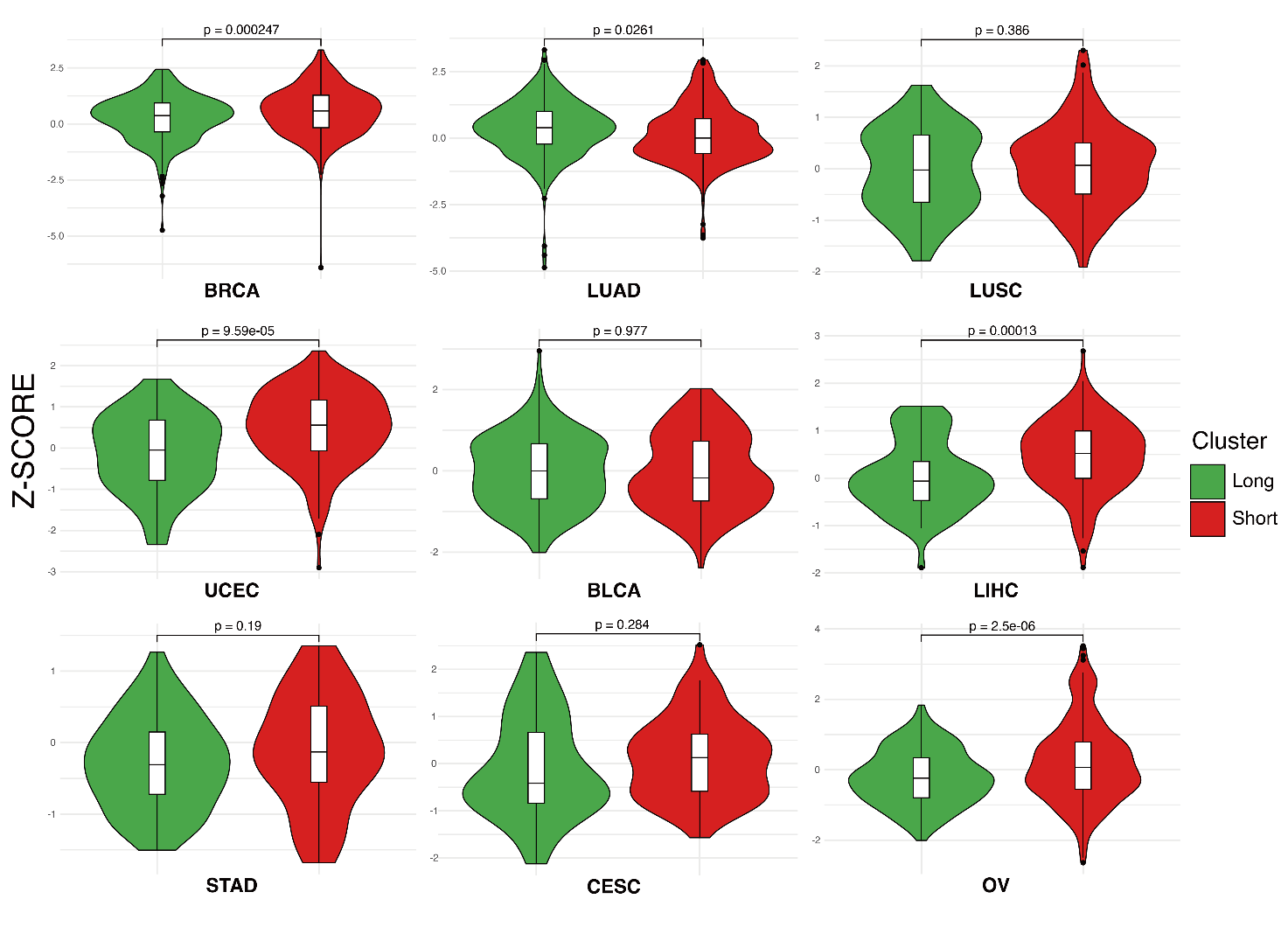


**Figure S7. General responsiveness of each prognosis-related groups of each cancer types**

The normalized z-score of all compounds were compared between short- and long-term risk groups of 9 cancer types cell lines. In LUAD and BLCA, the long-term risk groups showed higher resistance to perturbation with compounds, while in other cancer types the short-term risk groups were more resilient to compounds.


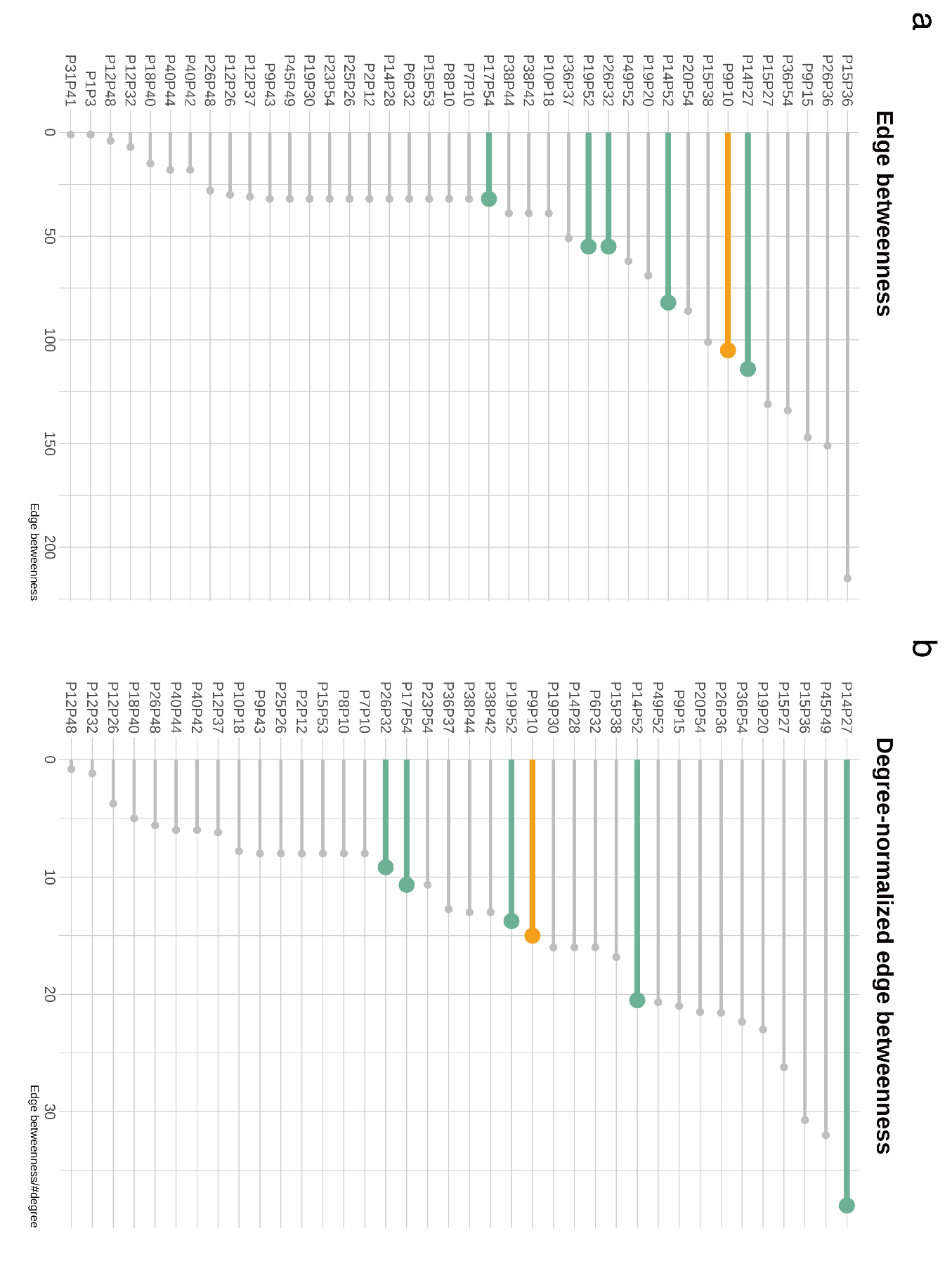


**Figure S8. Network structure properties of pathway crosstalks in STAD pathway interaction network**

For each node in the pathway interaction network of STAD, the edge betweenness was calculated. Edge betweenness is defined as the number of shortest paths passing through the given edge (left). Since this measurement is naturally skewed towards edges with high connections, it was normalized with the degree of the given edge (right).


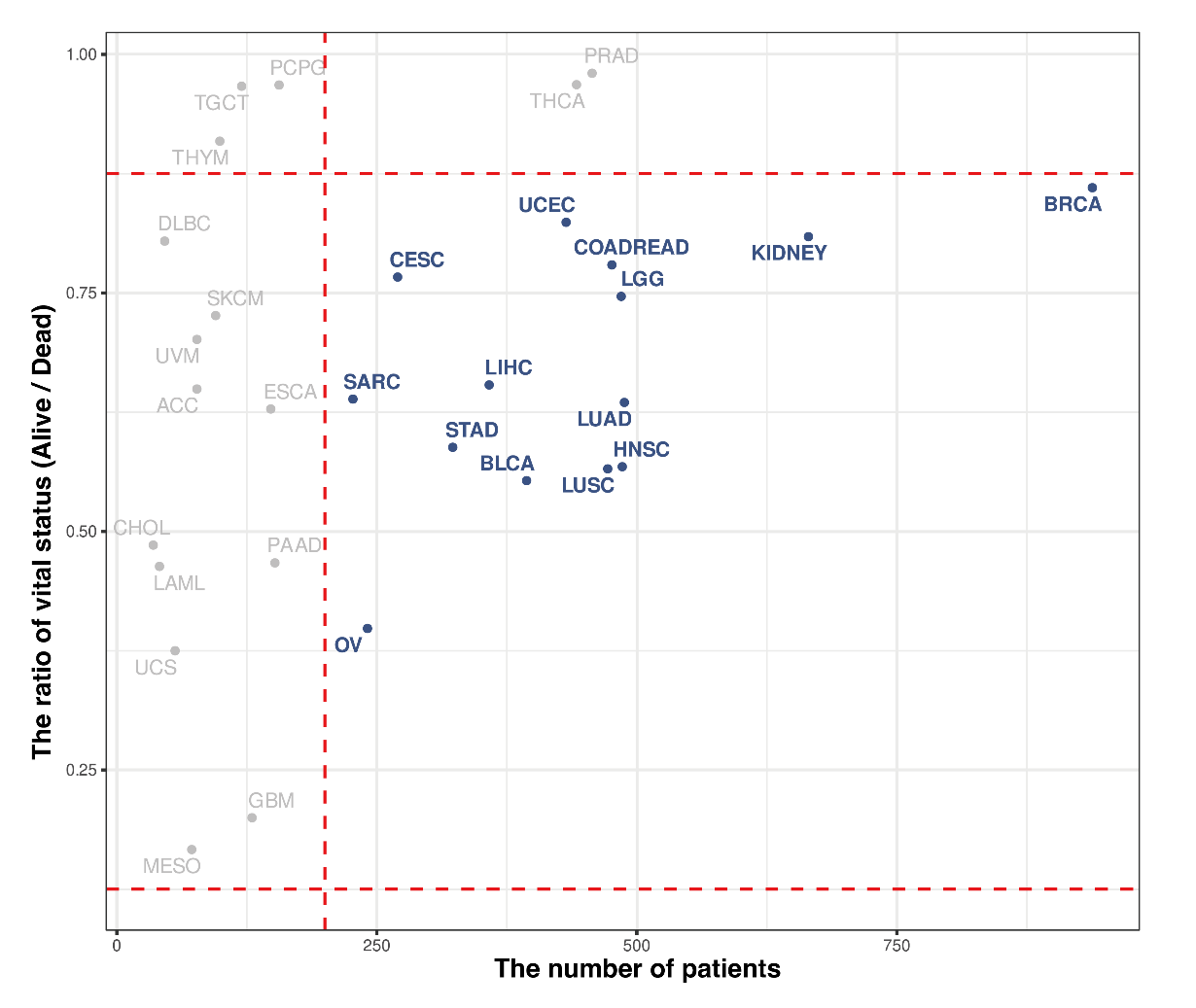


**Figure S9. Distribution of number of patients and the vital status ratio of TCGA cancer types**

The number and the dead/alive ratio of patients with cancer were both considered to select cancer types for NetG2P training and analysis. Only cancer types with patients count of 200 or more with a vital status ratio between 0.15 and 0.85 were selected (Dashed red line for cutoffs). For cancer types the TCGA abbreviation was used except for COADREAD, which combined COAD and READ, and KIDNEY, which consisted KICH, KIRC and KIRP.


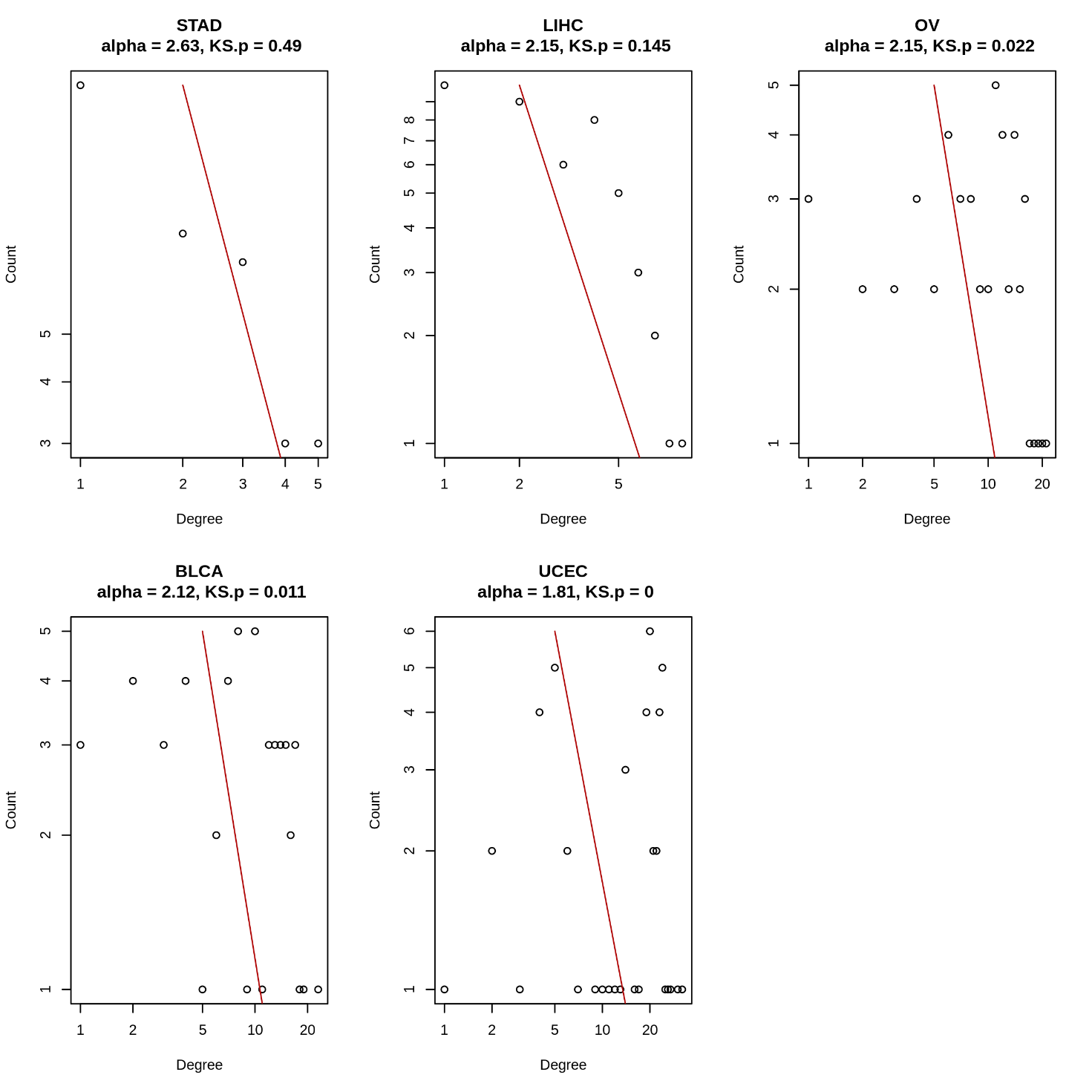
**. Figure S10. The node degree distribution of pathway interaction networks**

The pathway interaction networks constructed with COFs were analyzed for structural properties. The nodes with link number greater than zero were counted and the linear fit was performed. The p-value of the Kolmogorov-Smirnov test was used to determine the distribution fitting with a power law; a p-value > 0.05 indicated a power law fit, which was the case for STAD and LIHC. The alpha values of STAD and LIHC lay between 2 and 3.
