# Supplementary material for: NetG2P: Network-based genotype-to-phenotype transformation identifies key signaling crosstalk for prognosis in pan-cancer study
Source: BMC Biol. 2026 Feb 24;24:85. doi: 10.1186/s12915-026-02559-x (PMC13037077; doi:10.1186/s12915-026-02559-x)
Supplement: Supplementary file 4 — Additional file 4. Supplementary methods. NetG2P was compared with other network- and pathway-based methods to compare its performance in predicting vital status of cancer patients as well as predicting prognosis. The file describes detailed procedure for benchmarking configuration. [file 12915_2026_2559_MOESM4_ESM.docx]

**Supplementary Methods**

**Benchmarking**

To ensure a fair comparison, we kept the downstream feature construction (pathway feature generation) and ML pipeline identical wherever possible, and replaced only the upstream step that defines the method-specific per-patient gene sets/representations (NetG2P propagation + giant-cluster extraction) with each alternative method. All network-based methods used the same undirected PPIN backbone, and all methods used the same TCGA mutation data (and expression data where applicable). PROGENy is signature-based and does not require the PPIN; accordingly, we used PROGENy’s native pathway activity scores as features without the enrichment step used for gene-set–based methods.

• HotNet (Hierarchical HotNet):

We used the Hierarchical HotNet framework as a baseline, but adapted it to a patient-level setting to enable a fair, per-patient comparison with NetG2P. Hierarchical HotNet is originally designed for cohort-level subnetwork discovery rather than producing a patient-specific gene set. Therefore, for each patient we applied the HotNet/Hierarchical HotNet heat-diffusion step on the same PPIN using that patient’s mutated genes as seeds (implemented as random walk with restart / personalized PageRank; restart probability α = 0.5; maximum iterations = 100; convergence tolerance = 1×10⁻⁶). We then converted the resulting diffusion scores into a discrete patient-level gene set by selecting genes above a fixed threshold (top 10%, i.e., above the 90th percentile).

• DawnRank:

We used the DawnRank R package (v1.0) as a baseline and adapted it to match NetG2P’s per-patient input/output constraints. DawnRank generates a per-patient gene ranking; to enable a direct comparison to NetG2P’s per-patient gene-set representation, we converted each patient’s DawnRank ranking into a patient-specific driver gene set using a fixed threshold (top 10%, i.e., above the 90th percentile). This analysis was limited to 9 cancer types that have matched tumor-normal RNA-seq samples in TCGA (Supplementary Table R3). Three cancer types were excluded: OV and LGG have no matched normal RNA-seq samples in TCGA (0 cases), and CESC has only 2 matched pairs, which was insufficient for analysis.

**Table R3. TCGA Matched Normal Sample with Eligibility Criteria**

| Cancer_Type | GDC_Project | NetG2P_Samples | Normal_Cases | Matched_Pairs | Match_Rate | Eligible |
| --- | --- | --- | --- | --- | --- | --- |
| BLCA | TCGA-BLCA | 392 | 19 | 18 | 4.60% | PASS |
| STAD | TCGA-STAD | 318 | 36 | 29 | 9.10% | PASS |
| LUAD | TCGA-LUAD | 479 | 59 | 58 | 12.10% | PASS |
| LIHC | TCGA-LIHC | 357 | 50 | 46 | 12.90% | PASS |
| LUSC | TCGA-LUSC | 466 | 51 | 47 | 10.10% | PASS |
| BRCA | TCGA-BRCA | 937 | 113 | 103 | 11.00% | PASS |
| UCEC | TCGA-UCEC | 432 | 35 | 14 | 3.20% | PASS |
| COADREAD | TCGA-COAD, TCGA-READ | 475 | 51 | 34 | 7.20% | PASS |
| KIDNEY | TCGA-KIRC, TCGA-KIRP, TCGA-KICH | 663 | 129 | 119 | 17.90% | PASS |
| CESC | TCGA-CESC | 270 | 3 | 2 | 0.70% | NOT_PASS |
| OV | TCGA-OV | 240 | 0 | 0 | 0.00% | NOT_PASS |
| LGG | TCGA-LGG | 484 | 0 | 0 | 0.00% | NOT_PASS |

For each cancer type, we prepared three inputs required by DawnRank: (1) a PPI network as an undirected adjacency matrix, (2) patient-specific somatic mutation profiles as a binary mutation matrix, and (3) log2-transformed TPM expression data for both tumor and matched normal samples. We applied DawnNormalize to compute differential expression between each patient's tumor and matched normal tissue, then ran DawnRank with default parameters (damping factor μ = 3, maximum iterations = 100, convergence tolerance ε = 1×10⁻⁴). Genes above the 90th percentile were selected as each patient's driver gene set.

• TieDIE:

We applied a TieDIE-based bidirectional diffusion baseline and adapted it for per-patient analysis to enable fair comparison with NetG2P under identical input/output constraints. TieDIE is designed to identify linker genes connecting upstream perturbations to downstream transcriptional changes via network diffusion. For each patient, upstream seed genes were defined as somatically mutated genes, and downstream seed genes were defined as differentially expressed genes (z-score > 2 or < −2 compared to cohort mean). We performed personalized PageRank from both upstream and downstream seeds on the same undirected PPIN (alpha = 0.5, maximum iterations = 100). The combined diffusion score for each gene was calculated as the product of upstream and downstream PageRank scores, and genes above a fixed threshold (top 10%, i.e., above the 90th percentile) were selected as the patient-specific linker gene set for downstream feature construction.

• PROGENy:

We used PROGENy (Pathway RespOnsive GENes) to compute pathway activity scores from gene expression data using the decoupler Python package (v2.1.2). PROGENy estimates the activity of 14 signaling pathways based on consensus gene signatures derived from perturbation experiments. For each cancer type, we applied PROGENy to log2-transformed TPM expression data with default parameters (top=100 footprint genes per pathway, multivariate linear model method). Since PROGENy directly provides pathway activity scores, we used these 14 pathway scores as ML features without the hypergeometric enrichment test step applied to other methods.

NetG2P demonstrated the strongest performance among the compared methods in predicting patient vital status (Supplementary Figure R1 and Supplementary Table R4).

**Table R4. Benchmark scores against other network and pathway-based methods in predicting patients’ vital status**

| Method | F1-score (mean +/- std) | MCC (mean +/- std) | Accuracy (mean +/- std) |
| --- | --- | --- | --- |
| NetG2P | 0.892 +/- 0.048 | 0.668 +/- 0.087 | 0.868 +/- 0.035 |
| HotNet | 0.506 +/- 0.174 | 0.092 +/- 0.107 | 0.422 +/- 0.179 |
| DawnRank | 0.555+/- 0.351 | 0.141+/- 0.296 | 0.648+/- 0.208 |
| TieDIE | 0.491 +/- 0.162 | 0.066 +/- 0.083 | 0.405 +/- 0.167 |
| PROGENy | 0.490 +/- 0.152 | 0.136 +/- 0.066 | 0.465 +/- 0.100 |

Values are reported as mean ± SD across cancer types. For NetG2P, HotNet, TieDIE, and PROGENy, N = 12 cancer types were evaluated; for DawnRank, N = 9 cancer types were evaluated due to its requirement for matched tumor-normal RNA-seq.

Statistical analysis using paired Wilcoxon signed-rank tests on the per-cancer performance metrics confirmed that NetG2P significantly outperforms all other methods (p < 0.001 for all comparisons). We additionally provide per-cancer comparisons in Supplementary Figure R1. To avoid tuning, we used each method’s standard/default parameterization where applicable. For gene-ranking/score–based baselines that require conversion to a discrete per-patient gene set (HotNet-family diffusion, DawnRank, TieDIE-inspired), we applied a single pre-specified percentile rule (top 10%, i.e., 90th percentile) consistent with common practice and method recommendations, and did not tune this threshold to optimize performance.

We also conducted Kaplan–Meier (KM) survival analyses using COF values generated from these alternative methods. With HotNet, only three cancer types—BLCA, LGG, and COADREAD—showed statistically significant patient stratification. DawnRank did not yield statistically significant stratification. TieDIE produced significant results for two cancer types (LIHC and LUSC). Finally, PROGENy demonstrated significance in LGG.

**Note on HotNet implementation:** The reviewer mentioned both HotNet2 and Hierarchical HotNet. We note that the official HotNet2 GitHub repository (https://github.com/raphael-group/hotnet2) now directs users to Hierarchical HotNet as the latest and recommended version. In our benchmarking, we therefore used the HotNet/Hierarchical HotNet diffusion framework and adapted it to per-patient gene-set extraction (as described above) to match NetG2P’s per-patient input/output requirements.
